# Fragment‐Linked Purine Derivatives as Inhibitors of (p)ppGpp Synthetases: Design, Synthesis, and Structure‐Activity Relationships

**DOI:** 10.1002/ardp.70368

**Published:** 2026-10-26

**Authors:** Rayan Aboalgasim Makawi Abdalrahman, Katharina Sophie Erlitz, Joana Massa, Christopher‐Nils Mais, Gert Bange, Anna Junker, Oliver Koch

**Affiliations:** ^1^ Department of Preclinical Imaging and Radiopharmacy Werner Siemens Imaging Center, Cluster of Excellence iFIT (EXC 2180) ‘Image‐Guided and Functionally Instructed Tumor Therapies', University of Tuebingen Tuebingen Germany; ^2^ Institute of Pharmaceutical and Medicinal Chemistry University of Münster Münster Germany; ^3^ Center for Synthetic Microbiology (SYNMIKRO), Faculty of Chemistry, Microbes‐for‐Climate (M4C) Cluster of Excellence Philipps University Marburg Marburg Germany

**Keywords:** alarmone, antibiotics, (p)ppGpp, structure‐activity relationships

## Abstract

The bacterial stringent response, mediated by the alarmone nucleotides (p)ppGpp, plays a central role in persistence of infections, stress adaptation, and antimicrobial tolerance, making (p)ppGpp synthetases attractive targets for novel antibacterial strategies/therapies. In this study, we report the design, synthesis, and biological evaluation of fragment‐linked nucleobase derivatives targeting the RelA/SpoT homolog (RSH) enzymes. Guided by structural insights into adenine and guanine binding within small alarmone synthetase (SAS2/RelP), a series of purine‐based compounds was developed to simultaneously occupy conserved nucleotide‐binding regions. Initial linker‐bridged analogues showed limited activity, primarily due to poor solubility, prompting the introduction of ribose‐containing derivatives to improve physicochemical properties. Biochemical evaluation using HPLC‐based assays revealed selective inhibition of *Staphylococcus aureus* SAS1, with the most potent compound displaying micromolar activity (IC_50_ = 1.8 µM), while little or no inhibition was observed for related SAS enzymes. Structure‐activity relationship analysis highlights the importance of linker length and nucleobase positioning for enzyme selectivity. These findings establish fragment‐linked nucleobase scaffolds as a promising starting point for the development of inhibitors targeting (p)ppGpp biosynthesis and the bacterial stringent response.

## Graphical abstract

Fragment‐linked purine derivatives were designed to mimic nucleotide binding in bacterial (p)ppGpp synthetases. Structure‐guided optimization enabled selective inhibition of Staphylococcus aureus SAS1, revealing a new chemical strategy for targeting the stringent response and mechanisms of bacterial persistence.

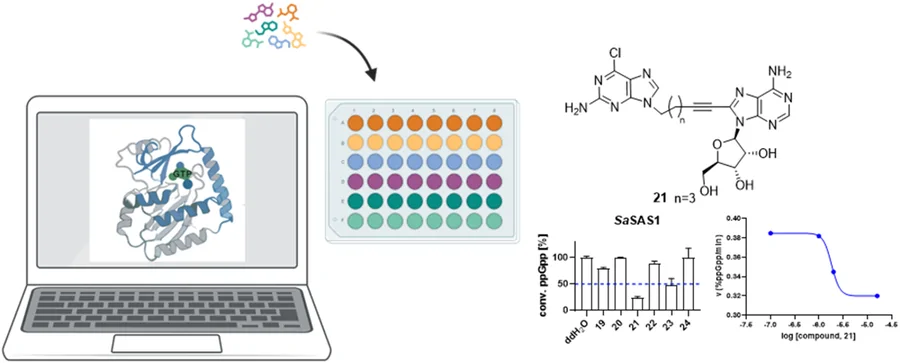

**Abbreviations**
AMP: adenosine‐5′‐monophosphate
AMR: antimicrobial resistance
ATP: adenosine‐5′‐triphosphate
BsSAS1: SAS1 from *Bacillus subtilis*
BsSAS2: SAS2 from *Bacillus subtilis*
calcd: calculated
d: days
DMF: *N*,*N*‐dimethylformamide
Et_3_N: triethylamine
EtOH: ethanol
GDP: guanosine‐5′‐diphosphate
h: hours
HEPES: 4‐(2‐hydroxyethyl)−1‐piperazineethanesulfonic acid
HPLC: high‐performance liquid chromatography
l: length of the column
melting point: Pd(PPh)_2_Cl_2_, bis(triphenylphosphine)palladium dichloride
mp, LC‐MS: liquid chromatography−mass spectrometry
(p)ppGpp: guanosine pentaphosphate (pppGpp) and guanosine tetraphosphate (ppGpp)
p‐TsOH.H_2_O: *p*‐toluenesulfonic acid monohydrate
R_*f*_
: retention factor
SAS: small alarmone synthetases
SaSAS1: SAS1 from *Staphylococcus aureus*
SaSAS2: SAS2 from *Staphylococcus aureus*

## 1 Introduction

The emergence of multidrug‐resistant bacteria represents a severe threat to global health. According to a study published in *The Lancet* in 2019, around 1.27 Mio deaths were a direct effect of antimicrobial resistance (AMR), and 4.95 Mio deaths were associated with bacterial AMR [1]. A steady decrease in effective broad‐spectrum antibiotics and the 40‐year innovation gap between 1962 and 2000 in the area of antibiotic research have led to a drastic increase in the number of multidrug‐resistant species [2]. The general understanding of antibiotic resistance is defined as the loss of the pathogen's susceptibility to an antibiotic after the antibiotic has reached the market [3]. Three central mechanisms, namely resistance, persistence, and tolerance, are leading to AMR. The emergence of resistance can be explained by mutations in important genes, such as the target gene, or by horizontal gene transfer of resistance factors from other bacteria. Other factors contributing to transient resistance include biofilm formation and bacterial persistence, which result in low susceptibility to various antibiotics. Stringent persistence has been identified in all major pathogens and is characterized by dormant or low‐activity cells that exhibit transient antibiotic tolerance. (p)ppGpp is an essential second messenger and signaling molecule of the stringent response and has been identified as a central regulator of persister cell formation. Synthesis and degradation of (p)ppGpp are mediated by enzymes of RelA/SpoT homology (RSH)‐type, which cover long RSH‐type enzymes and small alarmone synthetases (SAS) with structural and mechanistic differences [4]. Inhibition of (p)ppGpp synthesis by relacin (**1**, Figure 1a) was also shown to disrupt biofilm formation [5]. Inhibition of (p)ppGpp biosynthesis via RSH‐type enzymes is believed to have a harmful effect on bacterial survival. Recently, Tkachenko et al. [6] demonstrated that a synthetic diterpene analog inhibiting (p)ppGpp synthetases reduces persister cell formation, eradicates biofilms, and lowers cell survival. Therefore, the (p) ppGpp‐synthesizing RSH‐type enzymes represent the perfect protein family for a novel conceptual approach to developing species‐specific antibiotics and treating multidrug‐resistant pathogens.

**Figure 1:**
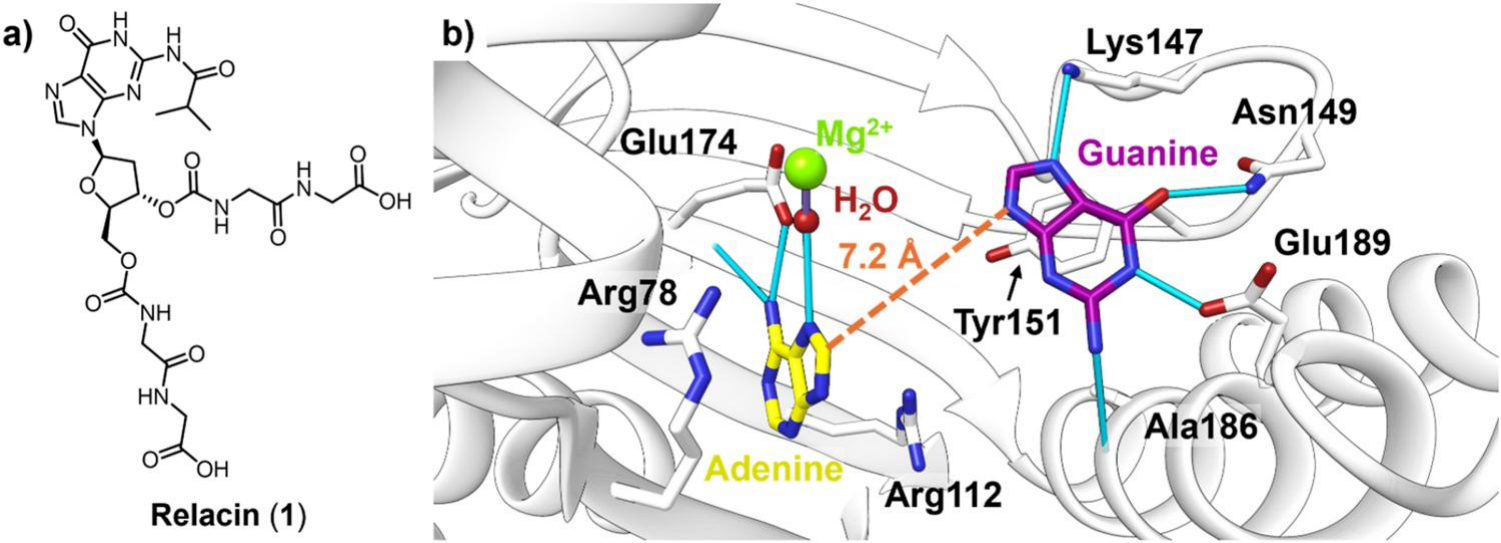
(a) Structure of the (p)ppGpp synthesis inhibitor relacin (**1**). (b) Binding mode of guanine and adenine in the active site of *Sa*SAS2. The figure is based on the experimental structure PDB ID 6EWZ [7]. Guanine is depicted in dark purple, while adenine is colored yellow. The protein is displayed in white as ribbons, and side chains of important residues are shown as sticks. Hydrogen bonds are depicted as light blue sticks, while the complex bond between water (red) and Mg^2+^ (green) is displayed in purple. The distance between the two nuclear bases was measured based on the coordinates of C8 (adenine) and N‐9 (guanine), which are the attachment points for the linkers of the analogs synthesized in this work.

In 2015 and 2018, the structural and mechanistic properties of the small (p)ppGpp synthetases RelQ (SAS1) and RelP (SAS2) were characterized [8, 9]. During (p)ppGpp synthesis, ATP nucleobase, adenine, and GDP nucleobase, guanine, interact with the conserved binding pocket of RelP of *Staphylococcus aureus* (SAS2). Adenine interacts with Arg78, Arg112, and Glu174, while guanine interacts with Lys147, Asn149, Tyr151, Ala186, and Glu189. The distance between the two nucleobases is 7.2 Å (Figure 1b). In more detail, the N‐7 of adenine forms a hydrogen bond with the oxygen of a Mg^2+^‐bound water. Furthermore, adenine interacts with Arg78 and Arg112 via π‐cation interactions, and the exocyclic amino group of adenine forms hydrogen bonds with the backbone and side chain carbonyl groups of Glu174. While guanine interacts via π‐stacking interactions with Tyr151, the amino group of Lys147 interacts with 7‐N of guanine via a hydrogen bond. The carbonyl group of guanine forms a hydrogen bond with the side chain nitrogen of Asn149. The exocyclic amino group of guanine interacts via hydrogen bonds with the backbone carbonyl of Ala186, as shown in Figure 1b [7, 9]. As the binding of guanine and adenine is assumed to be highly conserved in terms of interactions and spatial arrangement within the RSH enzymes, the aim of this study was to create potent inhibitors targeting the RSH‐type enzymes by blocking the respective binding pockets of adenine and guanine.

It can be assumed that guanine and adenine binding are highly conserved in terms of interactions and spatial arrangement since the underlying reactions have specific requirements that must exist in all (p)ppGpp synthetases, and therefore, a highly active inhibitor that targets all RSH‐type enzymes is feasible.

## 2 Results and Discussion

### 2.1 Chemistry

A fragment‐linking approach linking the two nucleobases was envisaged. The linker should permit the resulting molecule to represent the spatial conformation of GTP and ATP binding in the conserved binding site, actively blocking the binding site. Therefore, a simple carbon linker with lengths of 4 and 5 CH_2_ units was initially chosen based on the measured distance (Figure 1b).

Starting from 6‐chloro‐2‐aminopurine (**2**), the linker was introduced using the respective bromoalkyl ester (Scheme 1). Next, the respective ester **6** or **7** was reacted with various pyrimidine amines. Hereby, two different approaches were followed: reaction to the respective amides **8a**–**c** using Eaton's reagent following hydrolysis at the guanine moiety using aqueous NH_4_OH and two‐step formation of the amide and ring closure using a stronger base such as NaOH under reflux conditions, with various pyrimidine and pyridine amines to form the second heterocyclic system leading to the linked tested compounds **9a**–**q** (Scheme 2). Hereby, the substitution pattern was varied to investigate the effects of the placement of endocyclic nitrogens and the substituents at the 5‐position on inhibitory activity.

**Scheme 1:**
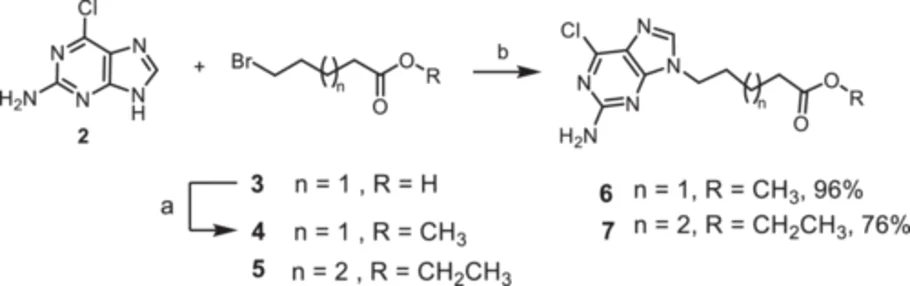
Reaction and conditions: (a) CH_3_OH, H_2_SO_4_, rt, 1 d; (b) K_2_CO_3_, NaI, DMF, rt, 5 d.

**Scheme 2:**
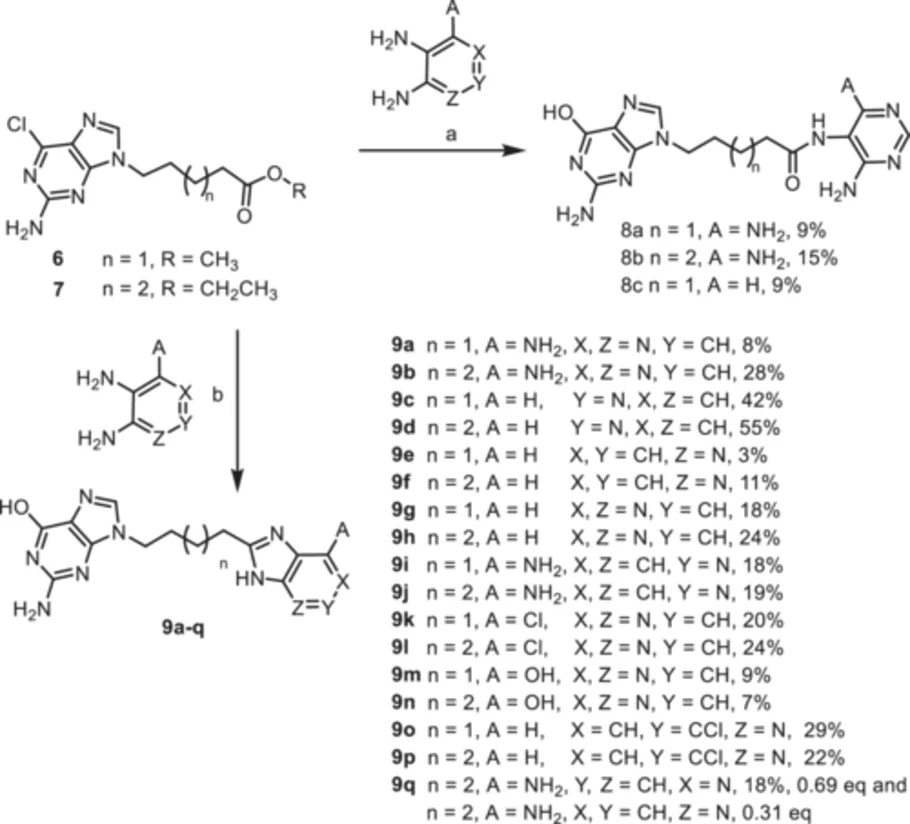
Reaction and conditions: (a) Eaton's reagent, 80°C, 3 h; NH_4_OH_aq._, 0°C, 30 min; (b) Eaton's reagent, 80°C, 3 h; NaOH, 100°C, 1 d; HCl_aq_, 0°C, 10 min.

However, since all derivatives suffered from low solubility, we considered introducing the ribose moiety to improve this parameter. Therefore, a combination of 6‐chloro‐2‐aminopurine (**2**) and adenosine (**17**) was envisaged. The linker bearing a terminal alkyne group was introduced onto 6‐chloro‐2‐aminopurine (**2**) using the respective bromoalkyne **10** or **11** via a nucleophilic substitution reaction (Scheme 3). Linkage to the adenosine at the 8‐position was then performed either via the Sonogashira reaction of protected 8‐bromoadenosine (**16**) or through Huisgen cycloaddition using 8‐azidoadenosine (**15**) (Scheme 4). Furthermore, 5′‐azido adenosine (**18**) was prepared to explore an alternative linkage position (Scheme 3). The linker length was again varied from 7 to 6 atoms for compounds **19** and **20** and 6 to 5 for compounds **21** and **22**, respectively. For compounds **23** and **24**, the linker was introduced at the 5'‐position of the ribose moiety (Scheme 4).

**Scheme 3:**
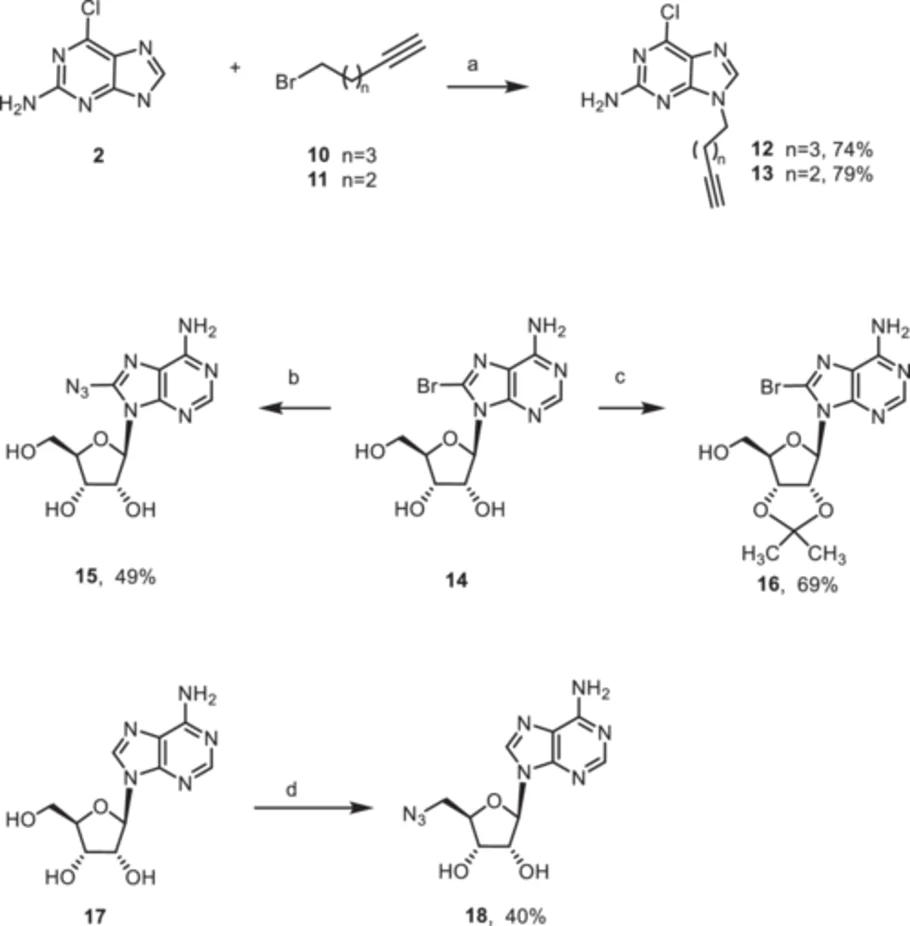
Reaction and conditions: (a) K_2_CO_3_, DMF, rt, overnight; (b) NaN_3_, DMF, 60°C, overnight; (c) *p*‐TsOH.H_2_O, 2,2‐dimethoxypropane, acetone, rt, 1 d; (d) SOCl_2_, pyridine, 0°C, 4 h; NaN_3_, DMF, 80°C, 5 h.

**Scheme 4:**
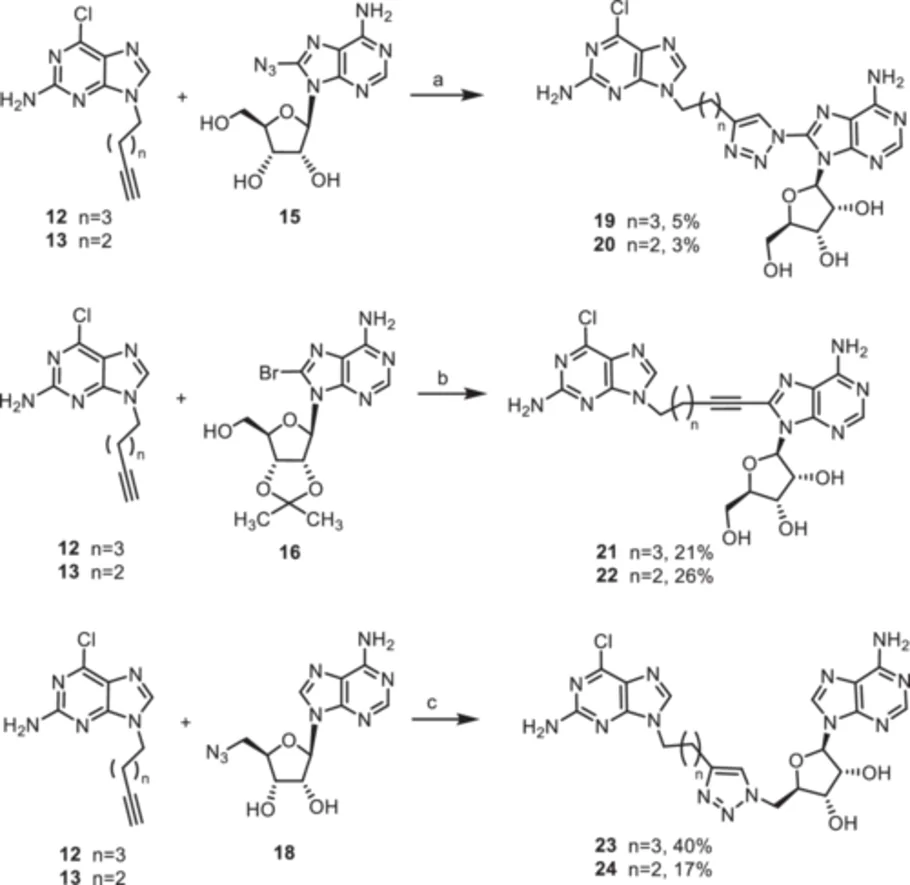
Reaction and conditions: (a) Cu(OAc)_2_, sodium ascorbate, EtOH, 50°C, 2 d; (b) 1. Pd(PPh)_2_Cl_2_, CuI, Et_3_N, DMF, 80°C, overnight; 2. NH_3_/CH_3_OH, 50°C, 2 h, 3. Dowex H, CH_3_OH/H_2_O (v/v 1:1), 50°C, 5 h; (c) Cu‐acetylacetonate, sodium ascorbate, EtOH, rt, 2 d.

### 2.2 In Vitro Pharmacology

There are two classes of RSH enzymes responsible for the (p)ppGpp synthesis: long RSH enzymes and short alarmone synthetase enzymes (SAS) [10]. Among SAS enzymes, RelQ (SAS1) and RelP (SAS2) are the best‐characterized enzymes and are found in a wide range of bacteria [11]. According to structural analysis, SAS1 and SAS2 proteins share about 50% amino acid sequence identity, but they differ in their activation by stress signals and functional roles [9]. Therefore, we planned to evaluate the inhibitory effect of the prepared tested compounds on both SAS1 and SAS2 via an HPLC inhibition assay. The HPLC inhibition assay was conducted using SAS1 from *Bacillus subtilis* (*Bs*SAS1), SAS1 from *Staphylococcus aureus* (*Sa*SAS1), SAS2 from *Bacillus subtilis* (*Bs*SAS2), and SAS2 from *Staphylococcus aureus* (*Sa*SAS2). *Bs*SAS1, *Sa*SAS1, *Bs*SAS1 and *Sa*SAS2 proteins were heterologously produced in *E. coli* BL21(DE3) and purified. The K_m_ values for GDP are 0.3 ± 0.2 mM for SaSAS2 and 1.7 ± 0.1 mM for BsSAS1 [9]. The proteins were then incubated with GDP, ATP, and the respective inhibitor. HPLC analysis was carried out to monitor the conversion of GDP and ATP to ppGpp and AMP and was plotted against a reaction without an inhibitor. Any compound displaying a promising inhibitory effect (> 50% inhibition at 2 mM) was submitted for the determination of a full concentration‐response curve.

At a 2 mM concentration of the tested compounds (**8a**–**8c**, **9a**–**9q**), no significant inhibition of ppGpp synthesis from *Bs*SAS1 was observed, except for compounds **8b** and **9b**, which could slightly inhibit the synthesis of ppGpp by around 34% and 23%, respectively (Figure 2a).

**Figure 2:**
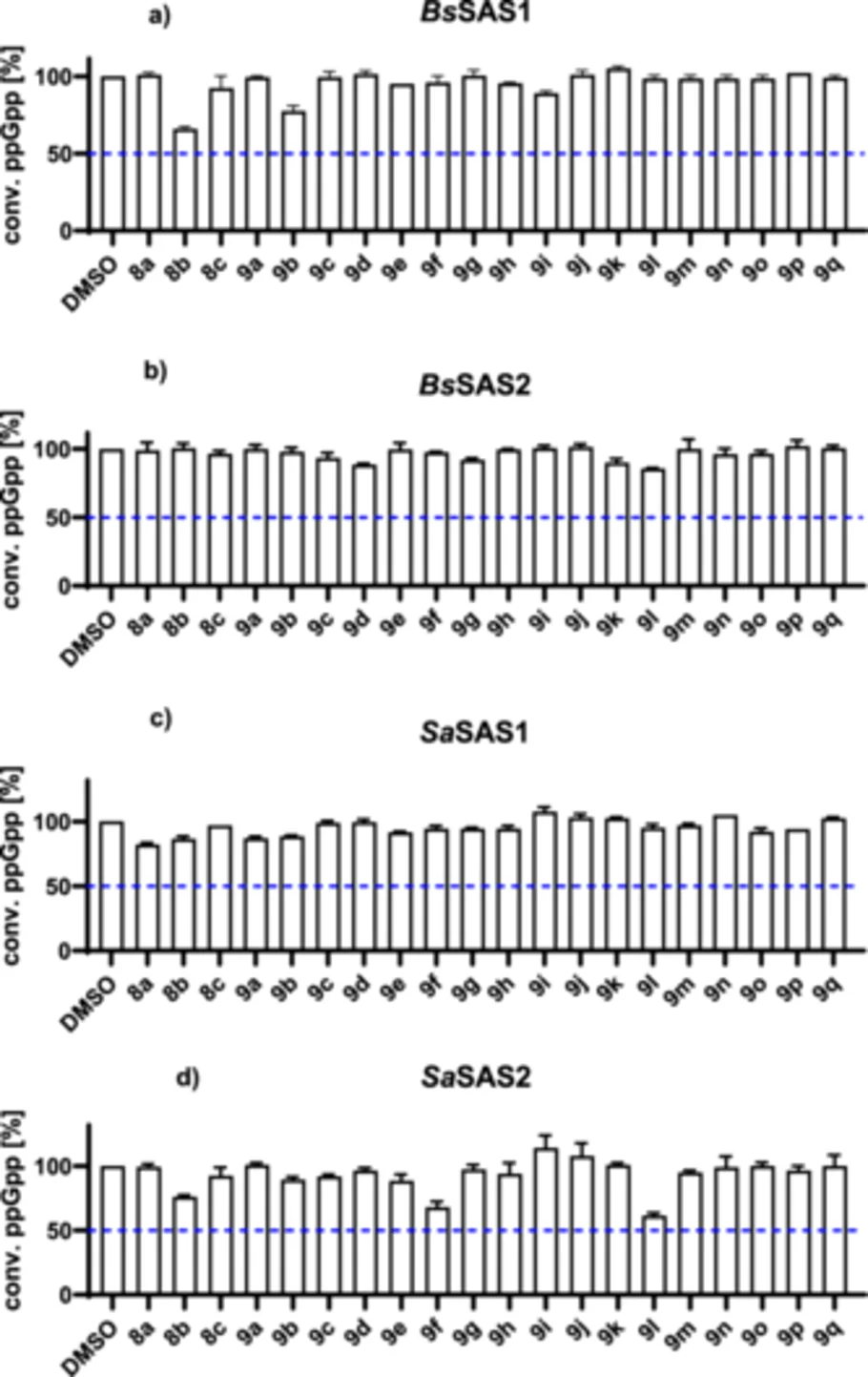
The inhibitory effecEffects of compounds **8a–8c** and **9a–9q** on ppGpp synthesis catalyzed by (a) SAS1 from Bacillus subtilis (*Bs*SAS1), (b) SAS2 from B. subtilis (*Bs*SAS2), (c) SAS1 from Staphylococcus aureus (*Sa*SAS1), and (d) SAS2 from S. aureus (*Sa*SAS2). Compounds were tested at 2 mM in the presence of 5 mM ATP and 1 mM GDP. Formation of ppGpp was quantified by HPLC and normalized to the DMSO control (100%). Data are presented as mean ± SD (*n* = 3). Blue dashed lines indicate 50% of control activity.

For *Bs*SAS2, none of the compounds was active against *Bs*SAS2 (Figure 2b). The activity of compounds **8a**–**8c**, **9a**–**9q** was also evaluated against *Sa*SAS1 and *Sa*SAS2. At a 2 mM concentration, no inhibition of ppGpp synthesis from *Sa*SAS1 was detected for any compound (Figure 2c), while three compounds, **8b**, **9f**, and **9l**, exhibited a slight inhibition of ppGpp synthesis from SaSAS2 by around 24%, 32%, and 39%, respectively (Figure 2d).

Due to low solubility observed for all initial derivatives, compounds **19**–**24** were designed and synthesized by introducing a ribose moiety to improve this physicochemical parameter. The resulting compounds were subsequently evaluated for their inhibitory activity against *Bs*SAS1, *Sa*SAS1, and *Sa*SAS2.

At a concentration of 1 mM, compounds **19**–**24** showed no inhibition of ppGpp synthesis from *Bs*SAS1, with the exception of compound **21**, which produced around 20% inhibition (Figure 3a).

**Figure 3:**
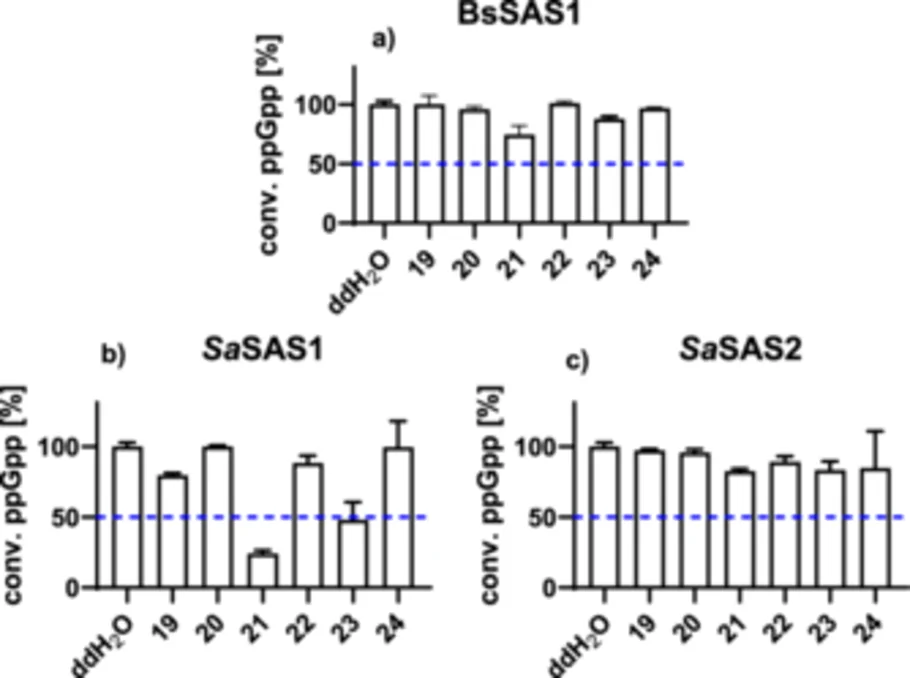
The Effects of compounds **19**–**24** on ppGpp synthesis catalyzed by (a) SAS1 from Bacillus subtilis (*Bs*SAS1), (b) SAS1 from Staphylococcus aureus (*Sa*SAS1), and (c) SAS2 from S. aureus (*Sa*SAS2). Compounds were tested at 1 mM in the presence of 1 mM ATP and 1 mM GDP. Formation of ppGpp was quantified by HPLC and normalized to the inhibitor‐free control (double‐distilled H_₂_O; 100%). Data are presented as mean ± SD (*n* = 3). Blue dashed lines indicate 50% of control activity.

For *Sa*SAS1, three compounds exhibited an inhibitory effect on ppGpp synthesis at a concentration of 1 mM. Compound **19** reduced ppGpp synthesis by around 20%, while compound **23** achieved a 52% inhibition. The most promising result was observed with compound **21**, which inhibited ppGpp synthesis by approximately 76% (Figure 3b). The activity of compounds **19**–**24** against *Sa*SAS2 was also evaluated (Figure 3c); however, none of the compounds was active (Figure 3c). Compounds **21** and **23** displayed significant inhibition of ppGpp synthesis in *Sa*SAS1 when compared to low or no inhibitory effects in *Bs*SAS1. Notably, although compounds **21** and **23** inhibited ppGpp synthesis against *Sa*SAS1 by approximately 80% and 50%, respectively, neither compound exhibited inhibitory activity against *Sa*SAS2.

As compound **21** demonstrated the most promising activity, its inhibitory activity against *Sa*SAS1‐mediated ppGpp synthesis was further evaluated in different concentrations, and samples were taken at different time points to determine the ppGpp synthesis from GDP and ATP per enzyme per min and to calculate the IC_50_ value. Compound **21** inhibited ppGpp synthesis by SaSAS1 with a pIC_50_‐value of 5.74 (IC_50_ = 1.8 µM) (Figure 4).

**Figure 4:**
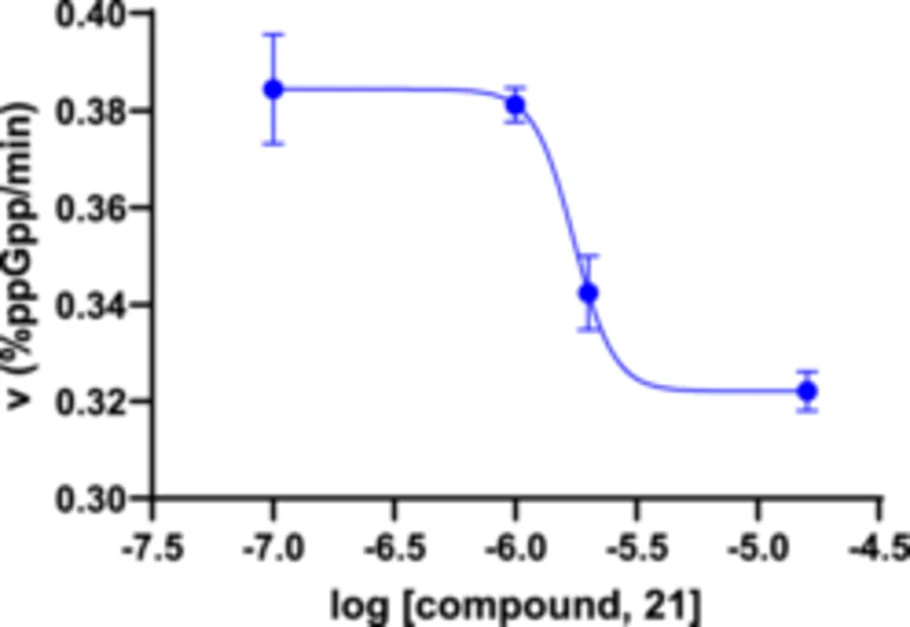
Concentration‐response curve of compound **21**. v (%ppGpp/min) at given **21** concentrations. v (%ppGpp/min) is measured as the production of ppGpp from GDP and ATP per enzyme per min ± SD (pooled data, *n* = 3).

The two compound series were evaluated under different screening conditions: compounds **8a**–**c** and **9a**–**q** were tested at 2 mM in the presence of 5 mM ATP, whereas compounds **19**–**24** were tested at 1 mM in the presence of 1 mM ATP. The lower ATP concentration was chosen for the second series to increase screening sensitivity to potential ATP‐competitive inhibitors and facilitate the identification of starting points for further optimization. Although the fragment‐linking design envisaged occupation of both nucleotide‐binding regions, the mechanism of inhibition remains to be established experimentally. If inhibition involves competition with ATP, the higher ATP concentration used for the first series could have attenuated the observed activity. Accordingly, both poor solubility and substrate competition may have contributed to the limited inhibition observed for these compounds. The different assay conditions preclude a direct comparison of potency between the two series; nevertheless, the second‐series screen identified compound **21** as a promising inhibitor of SaSAS1 under the conditions investigated.

### 2.3 Computational Modeling

This discrepancy in activity could be attributed to variations in the amino acid sequence of SAS1 across different bacterial species [7], or the difference in the activity of the two enzymes. As previously mentioned, SAS1 and SAS2 have 50% similarity in their amino acid sequences, but they exhibit differences in their activation and function. Unlike *Sa*SAS1, a metal ion–binding site has been identified in *Sa*SAS2, which contributes to its activity. The ability of *Sa*SAS2 to trigger a stringent response and enhance (p)ppGpp production appears to improve with the presence of Zn^2+^ ions, but the mode of action was unclear [7]. The loss of activity of the tested compounds in *Sa*SAS2 may be attributed to the reduced enzyme activity due to the absence of Zn^2+^ ions. Additionally, SAS1 and SAS2 display differences in the conformational properties of the guanosine‐substrate binding site, known as the G‐Loop. The G‐loop of SAS1 is highly disordered, while it is highly ordered in SAS2. This distinction is likely due to the presence of proline in SAS2, which contributes to the G‐Loop's rigidity, while it is absent in SAS1, resulting in a disordered G‐Loop (Figure 5) [9]. This variation in structure may influence how the inhibitors bind to SAS1 and SAS2, leading to different inhibitory effects since our compounds were designed based on the binding of GTP and ATP to the SAS enzymes.

**Figure 5:**
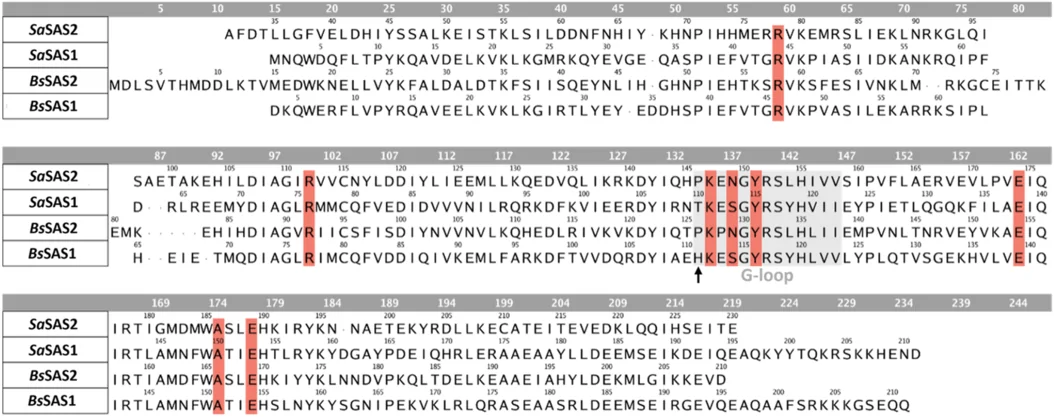
Sequence alignment of *Sa*SAS1, *Sa*SAS2, *Bs*SAS1, and *Bs*SAS2. Binding site residues are highlighted in orange and the G‐loop is marked in grey. The missing proline in SAS2 is marked with an arrow.

To gain further insights into the activity of the synthesized compounds, the binding mode of **21** was first modeled in *Sa*SAS2. The in silico results suggest that the adenine and guanine of **21** are able to retain most of the interactions observed for the unlinked nucleotide bases. As shown in Figure 6, the guanine‐like moiety of **21** forms hydrogen bonds with its N9 and exocyclic N with Lys147 and Glu189, respectively. The π‐cation and π–π‐interactions of the guanine‐like moiety and adenine with Arg78, Arg112, and Tyr151 are retained while the ribose moiety forms further hydrogen bonds with Arg78 and Arg112. However, the modification of the guanine carbonyl to a chloride leads to the loss of the hydrogen bond with Asn149. A single difference in the interacting amino acids was observed for the SAS2 and SAS1 enzymes (Figure 6b–d). While *Sa*SAS2 and *Bs*SAS2 contain an asparagine, this residue is serine in *Sa*SAS1 and *Bs*SAS1. All synthesized ligands feature a chloride atom in 6‐position, which, as discussed, hinders the hydrogen bond with Asn149. A serine in the same position takes up less space, and therefore the protein might more easily accommodate the bulkier chloride atom. Together with the increased flexibility of the G‐loop in SAS1, this explains the lack of activity of the synthesized compounds in SAS2 enzymes.

**Figure 6:**
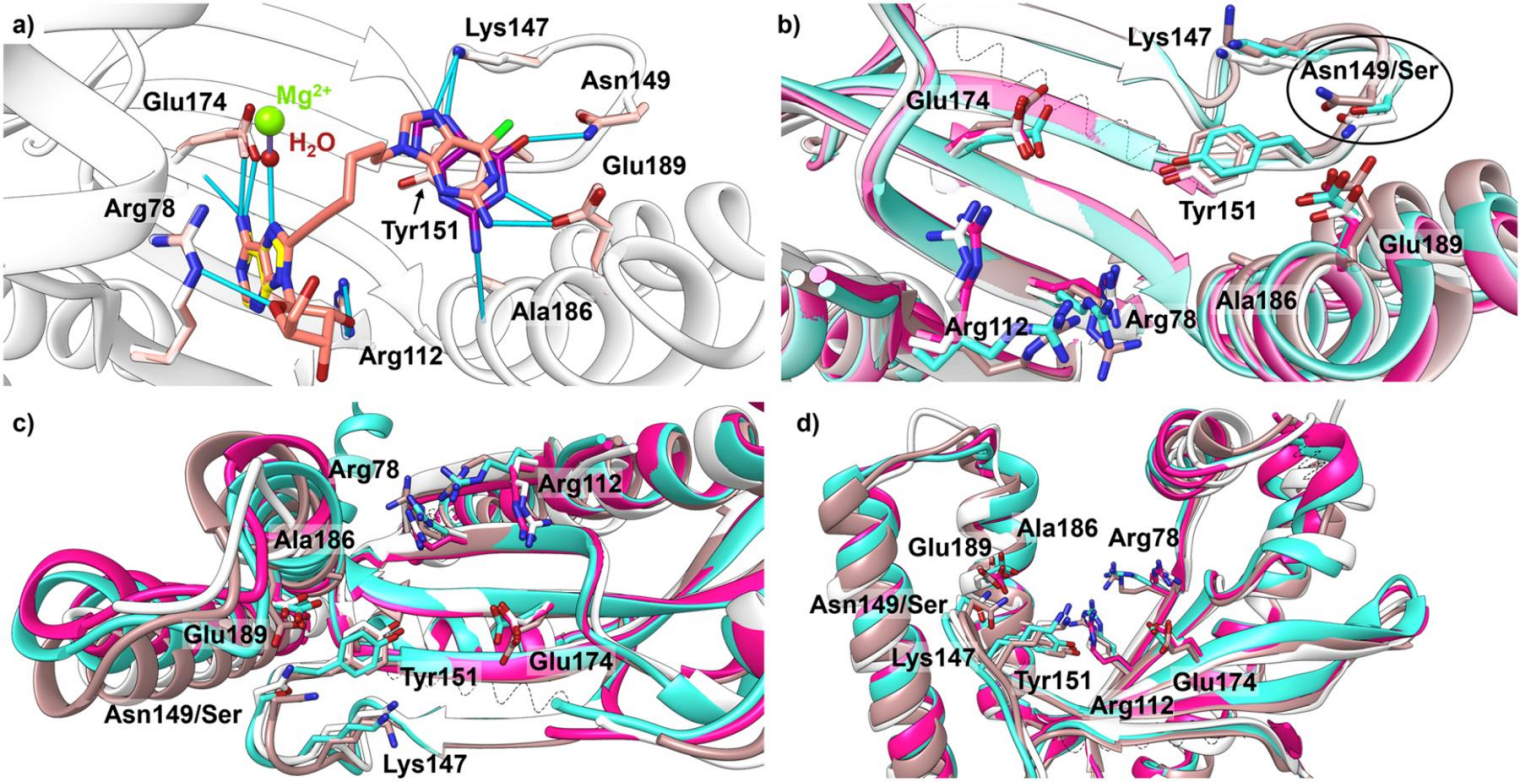
(a) Binding mode of 21 (orange) in *Sa*SAS2 (white) compared with the binding mode of guanine (purple) and adenin. Computational analysis of compound 21 binding and comparison of SAS nucleotide‐binding pockets. (a) Predicted binding pose of compound 21 (orange) in *Sa*SAS2 (white), superimposed on the reference guanine (purple) and adenine (yellow) moieties shown in Figure 1b. (b–d) Different views of the superimposed binding pockets of *Sa*SAS1 (cyan), *Sa*SAS2 (white), *Bs*SAS1 (pink), and *Bs*SAS2 (bronze), illustrating the spatial arrangement of corresponding binding‐site residues, including the Asn‐to‐Ser substitution between SAS2 and SAS1. Proteins are shown as ribbons, with selected side chains and ligands represented as sticks. The unresolved *Bs*SAS2 loop corresponding to the region containing Lys147, Asn149, and Tyr151 in SaSAS2 is indicated by a dotted black line. Hydrogen bonds are shown in light blue, and coordination between water (red) and Mg²⁺ (green) is shown in purple.

For *Sa*SAS1, **19**, **21**, and **23** showed some to promising inhibitory activity. All these molecules have a linker with three carbon atoms, while their counterparts **20**, **22**, and **24** are less effective. This is consistent with the modeled binding pose of **21** in *Sa*SAS2, as a linker length of *n* = 2 is too short to allow the guanine‐like moiety and adenosine to retain their interactions observed in the experimental structure.

## 3 Conclusion

In this study, we designed and synthesized fragment‐linked purine derivatives as potential inhibitors of bacterial (p)ppGpp synthetases. The initial linker‐bridged nucleobase analogs showed only limited inhibitory activity, most likely due to unfavorable physicochemical properties, especially poor solubility. The introduction of a ribose moiety improved compound design and led to the identification of compound **21** as the most promising inhibitor, with selective inhibition of *Sa*SAS1‐mediated ppGpp synthesis and an IC_50_ of 1.8 µM.

The biological data indicate that inhibition is strongly dependent on linker length, nucleobase positioning, and the targeted SAS enzyme. Compounds with the longer linker, in particular **19**, **21**, and **23**, showed higher activity against *Sa*SAS1 than their shorter analogs, whereas no relevant inhibition was observed for *Sa*SAS2. Computational modeling suggests that compound **21** can retain key interactions of the natural adenine and guanine substrates within the binding pocket. The observed selectivity for SAS1 over SAS2 may be explained by structural differences in the binding site, including the Asn‐to‐Ser exchange and the higher flexibility of the SAS1 G‐loop, which may better accommodate the 6‐chloro‐substituted guanine‐like moiety of compound **21**.

Overall, these findings demonstrate that fragment‐linking of nucleobase motifs is a viable strategy for targeting bacterial alarmone synthetases. Although further optimization is required to improve potency, solubility, and broader antibacterial relevance, compound **21** provides a promising starting point for the development of selective inhibitors of (p)ppGpp biosynthesis and for further exploration of the bacterial stringent response as an antibacterial target.

## 4 Experimental

### 4.1 Chemistry

#### 4.1.1 General

Unless otherwise noted, moisture‐sensitive reactions were conducted under dry nitrogen. Flash column chromatography (fc): Silica gel 60, 40–64 µm; parentheses include: diameter of the column, length of the column, eluent, R_f_ value. Melting point: melting point apparatus Mettler Toledo MP30, uncorrected. IR: IR spectrophotometer FT‐ATR‐IR (Shimadzo®IRAffinity‐1S®). ^1^H NMR (400 MHz): Unity Mercury Plus 400 spectrometer (Varian®), AV400 (Bruker®), JEOL JNM‐ECA‐400. ^13^C NMR (100 MHz): Unity Mercury plus 400 spectrometer (Varian®) JEOL JNM‐ECA‐400; δ in ppm relative to tetramethylsilane; coupling constants are given with 0.5 Hz resolution, the assignments of ^13^C and ^1^H NMR signals were supported by 2D NMR techniques; MS: APCI = atmospheric pressure chemical ionization, EI = electron impact, ESI = electro‐spray ionization: MicroTof (Bruker Daltronics, Bremen), calibration with sodium formate clusters before measurement. All solvents were of analytical grade quality and demineralized water was used. HPLC solvents were of gradient‐grade quality, and ultrapure water was used. All HPLC eluents were degassed by sonication prior to use. Thin‐layer chromatography was conducted with silica gel F_254_ on aluminum plates in a saturated chamber at room temperature. The spots were visualized using UV light (254 nm) or reagents such as cerium molybdate dipping bath with additional heating using a standard heat gun. Hence, the retention factor values strongly depend on the temperature, the chamber saturation, and exact ratio of components of the eluent (highly volatile); the given retention factor values represent just approximate values. For purification of the compounds, a flash column chromatography with silica gel 600 (40–63 μm, Macherey‐Nagel), an automated flash chromatography: Biotage® Selekt with either prepacked C18 reversed phased‐ or normal phase silica columns, and preparative HPLC (Shimadzu HPLC system) were used. Applied solvent composition and gradient are detailed in the synthetic procedures. Lyophilization was performed by a Labconco® FreeZone® freeze dryer with a Hybrid vacuum pump.

#### 4.1.2 Semi‐Preparative HPLC

The main applied method (method C18) is shown. If changes to the purification method were made, details are given in the synthetic procedure. Equipment: UV‐detector: UltiMate VWD‐3400RS variable Wavelength Detector; manual sampler injector valve for UltiMate 3000 (800 µL sample loop); pump: UltiMate LPG‐3400SD; degasser: UltiMate LPG‐3400SD; fraction collector: UltiMate AFC‐3000; data acquisition: Chromeleon Client 6.8.0 (Dionex Corpor.). injection volume: 200–700 µL; detection at λ = 210, 230, 254 nm; column: Agilent technologies, Polaris ^tm^ C18‐A, 250 × 21.2 mm, 5 µm, precolumn: Agilent technologies, Polaris ^tm^ C18‐A, 30 × 21.2 mm, 5 µm, at room temperature. Solvent: A: CH_3_CN, B: H_2_O (milli Q), gradient elution: (A %): 0–2 min: 10%, flow 2 mL/min, 2–10 min: 10%, flow 5 mL/min, 10–40 min: gradient from 10% to 25%, 40–41 min: gradient from 25% to 100%, 41–55 min: 100%, 55–56 min: gradient from 100% to 10%, 56–66 min: 10%.

#### 4.1.3 HPLC Purity Measurements

*Method A:* Equipment: UV‐detector: UltiMate 3000 variable Wavelength Detector; autosampler: UltiMate 3000; pump: Ultimate 3000; degasser: Ultimate 3000; data acquisition: Chromeleon Client 8.0.0 (Dionex Corpor.). Method: column: guard column: Zorbax SB‐Aq 12.5 × 4.6 mm cartridge, column: Zorbax SB‐Aq StableBond analytical 150 × 4.6 mm, flow rate: 1.00 mL/min; injection volume: 5.0 μL; detection at λ = 210 nm; solvents: A: Tetrabutylammonium phosphate buffer (5 mM) in H_2_O, B: CH_3_CN, gradient elution: (A %): 0–20 min: gradient from 80% to 20%, 20–30 min: gradient from 20% to 80%.

*Method B:* Equipment: UV‐detector: UltiMate 3000 variable Wavelength Detector; autosampler: UltiMate 3000; pump: Ultimate 3000; degasser: Ultimate 3000; data acquisition: Chromeleon Client 8.0.0 (Dionex Corpor.). Method: column: guard column: Zorbax SB‐Aq 12.5 × 4.6 mm cartridge, column: Zorbax SB‐Aq StableBond analytical 150 × 4.6 mm, flow rate: 1.00 mL/min; injection volume: 5.0 μL; detection at λ = 210 nm; solvents: A: Tetrabutylammonium phosphate buffer (5 mM) in H_2_O, B: CH_3_CN, gradient elution: (A %): 0–20 min: gradient from 100% to 60%, 20–30 min: gradient from 60% to 100%.

*Method C:* DIONEX Ultimate 3000, UV detector: vwd‐3400 RS; autosampler: WPS‐3000 PL; pump: LPG‐3600, degasser: DG‐1210; data acquisition: Chromeleon 7 (Thermo Fisher Scientific). Method: guard column: LiChropher® 60 RP‐select B (5 μM), 4 × 4 mm cartridge, column: LiChrospher® 60 RP‐select B (5 μm), 250 × 4 mm^2^ column, flow rate: 1.00 mL/min; injection volume: 5.0 μL; detection at λ = 210 nm; solvents: A: H_2_O with 0.05% (v/v) trifluoroacetic acid; B: CH_3_CN with 0.05% (v/v) trifluoroacetic acid: gradient elution: (A %): 0–4 min: 90%, 4–29 min: gradient from 90% to 0%, 29–31 min: 0%, 31–31.5 min: gradient from 0% to 90%, 31.5–40 min: 90%.

All compounds are ≥ 95% pure by HPLC analysis.

#### 4.1.4 Synthesis of Methyl 5‐Bromopentanoate (4)

5‐Bromopentanoic acid **3** (2.00 g, 11.0 mmol, 1.0 eq.) was dissolved in CH_3_OH (20 mL) and a catalytic amount of H_2_SO_4_ (96%, 0.2 mL) was added. The mixture was allowed to stir at room temperature overnight, and the reaction was afterward stopped by the addition of saturated aqueous NaHCO_3_ (10 mL). The pH value was adjusted to 9 by the addition of aqueous NaOH (2 M), and the mixture was diluted with 20 mL of distilled H_2_O. The product was then extracted by diethyl ether (2 × 10 mL), and the combined organic layers were dried over Na_2_SO_4_. Ether was removed carefully under reduced pressure. After drying at low vacuo for a short period of time, the product was obtained as an orange liquid (1.89 g, 9.7 mmol, 88%). Purity (HPLC *A*): 100% (*R*_*t*_ = 10.7 min). Exact mass (ESI): m/z = calcd. for C_6_H_12_^79^BrO_2_ [M + H^+^] 195.0015, found 195.0025. ^1^H NMR (600 MHz, DMSO‐*d*_6_): δ (ppm) = 3.59 (s, 3H, C*H*_3‐methyl_), 3.53 (t, *J* = 6.7 Hz, 2H, 5‐C*H*_2‐pentyl_), 2.35 (m, 2H, 2‐C*H*_2‐pentyl_), 1.81 (m, 2H, 4‐C*H*_2‐pentyl_), 1.64 (m, 2H, 3‐C*H*_2‐pentyl_). ^13^C NMR (151 MHz, DMSO‐*d*_6_): δ (ppm) = 173.1 (*C*=O_ester_), 51.3 (*C*_methyl_), 34.6 (*C*−5_pentyl_), 32.3 (*C*−2_pentyl_), 31.5 (*C*−4_pentyl_), 23.1 (*C*−3_pentyl_). FTIR (neat): $\tilde{v}$ (cm^−1^) = 2951 (C─H), 1732 (C=O).

#### 4.1.5 Synthesis of Methyl 5‐(2‐Amino‐6‐Chloro‐9*H*‐Purin‐9‐yl)Pentanoate (6)

6‐Chloro‐9*H*‐purin‐2‐amine **2** (1.37 g, 8.1 mmol, 1.0 eq.) and potassium carbonate (2.91 g, 21.0 mmol, 2.0 eq.) were suspended in dry DMF (70 mL). The mixture was allowed to stir for 2 min, then methyl 5‐bromopentanoate **4** (2.05 g, 10.5 mmol, 1.3 eq.) in dry DMF (5 mL) and NaI (315 mg, 2.1 mmol, 0.2 eq.) were added. After stirring the mixture at room temperature for 4 days, the reaction was stopped by the addition of cold H_2_O (40 mL). The aqueous layer was extracted with CH_2_Cl_2_ (4 × 100 mL), combined organic layers were washed with brine, dried over Na_2_SO_4_ and the solvent was removed under reduced pressure. Residue was purified by flash column chromatography (cyclohexane/ethyl acetate = 90:10; ∅ = 5 cm, *l* = 12 cm) and product **6** was obtained as white solid (2.09 g, 7.7 mmol, 96%). *R*_*f*_ = 0.46 (10/90 cyclohexane/ethyl acetate). mp = 82°C. Purity (HPLC *A*): 99% (*R*_*t*_ = 6.3 min). Exact mass (ESI): m/z = calcd. for C_11_H_15_^35^ClN_5_O_2_ [M + H^+^] 284.0909, found 284.0914. ^1^H NMR (600 MHz, DMSO‐*d*_6_): δ (ppm) = 8.14 (s, 1H, 8‐C*H*_purine_), 6.90 (s, 2H, N*H*_2‐purine_), 4.05 (t, *J* = 7.0 Hz, 2H, 5‐C*H*_2‐pentyl_), 3.56 (s, 3H, 1‐C*H*_3‐methyl_), 2.33 (t, *J* = 7.4 Hz, 2H, 2‐C*H*_2‐pentyl_), 1.78 (m, 2H, 4‐C*H*_2‐pentyl_), 1.47 (m, 2H, 3‐C*H*_2‐pentyl_). ^13^C NMR (151 MHz, DMSO‐*d*_6_): δ (ppm) = 173.0 (*C* = O), 159.8 (*C*−2_purine_), 154.1 (*C*−4_purine_), 149.3 (*C*−6_purine_), 143.3 (*C*−8_purine_), 123.3 (*C*−5_purine_), 51.2 (*C*_methyl_), 42.6 (*C*−5_‐pentyl_), 32.6 (*C*−2_pentyl_), 28.3 (*C*−4_pentyl_), 21.4 (*C*−3_pentyl_). FTIR (neat): $\tilde{v}$ (cm^−1^) = 3213, 3318, 3117, 2959 (C─H/N─H), 1724 (C=O), 1609, 1558 (C=N).

#### 4.1.6 Synthesis of Ethyl 6‐(2‐Amino‐6‐Chloro‐9*H*‐Purin‐9‐yl)Hexanoate (7)

6‐Chloro‐9*H*‐purin‐2‐amine **2** (5.83 g, 34.5 mmol, 1.0 eq.) and potassium carbonate (12.39 g, 89.64 mmol, 2.0 eq.) were suspended in dry DMF (100 mL). Ethyl 6‐bromohexanoate **5** (10.00 g, 44.8 mmol, 1.3 eq.) and NaI (1.03 g, 6.9 mmol, 0.2 eq.) were added. After stirring the mixture at room temperature for 4 days, solvent was removed under reduced pressure. H_2_O (70 mL) was added and the product **7** was extracted with CH_2_Cl_2_ (4 × 100 mL), combined organic layers were washed with brine, dried over Na_2_SO_4_ and the solvent was removed under vacuo. Purification was carried out by flash column chromatography (cyclohexane/ethyl acetate = 40:60; ∅ = 8 cm, *l* = 10 cm) and product **7** was obtained as white solid (8.16 g, 26.2 mmol, 76%). *R*_*f*_ = 0.32 (40/60 cyclohexane/ethyl acetate). mp = 85°C. Purity (HPLC *B*): 100% (*R*_t_ = 9.0 min). Exact mass (ESI): m/z = calcd. for C_13_H_19_^35^ClN_5_O_2_ [M + H^+^] 312.1222, found 312.1217. ^1^H NMR (600 MHz, DMSO‐*d*_6_): δ (ppm) = 8.13 (s, 1H, 8‐C*H*_purine_), 6.89 (s, 2H, N*H*_2‐purine_), 4.03 (t, *J* = 7.2 Hz, 2H, 6‐C*H*_2‐hexyl_), 4.01 (q, *J* = 7.0 Hz, 2H, C*H*_2‐ethyl_) 2.26 (t, *J* = 7.3 Hz, 2H, 2‐C*H*_2‐hexyl_), 1.77 (p, *J* = 7.3 Hz, 2H, 5‐C*H*_2‐hexyl_), 1.53 (p, *J* = 7.4 Hz, 2H, 3‐C*H*_2‐hexyl_), 1.24 (m, 2H, 4‐C*H*_2‐hexyl_), 1.14 (t, 3H, *J* = 7.1 Hz, 1‐C*H*_3‐ethyl_). ^13^C NMR (151 MHz, DMSO‐*d*_6_): δ (ppm) = 172.8 (*C* = O), 159.8 (*C*−2_purine_), 156.8, and 154.5 (*C*−2_purine_ and *C‐*6_purine_), 154.1 (*C*−4_purine_), 149.3 (*C*−6_purine_), 143.3 (*C*−8_purine_), 123.3 (*C*−5_purine_), 59.7 (*C*−1_ethyl_), 42.8 (*C*−6_hexyl_), 33.3 (*C*−2_hexyl_), 28.6 (*C*−5_hexyl_), 25.4 (*C*−4_hexyl_), 23.9 (*C*−3_hexyl_), 14.1 (*C*−2_ethyl_). FTIR (neat): $\tilde{v}$ (cm^−1^) = 3422, 3318, 3202 (O─H/N─H), 2936, 2858 (C─H), 1728 (C=O), 1558 (C=N).

#### 4.1.7 Synthesis of 5‐(2‐Amino‐6‐Hydroxy‐9*H*‐Purin‐9‐yl)‐*N*‐(4,6‐Diaminopyrimidin‐5‐yl)Pentanamide (**8a**)

Compound **6** (300 mg, 1.1 mmol, 1.0 eq.) was dissolved in eaton's reagent at 80°C under inert conditions and the solution was stirred for 5 min. Pyrimidine‐4,5,6‐triamine (167 mg, 1.3 mmol, 1.2 eq.) was added and mixture was stirred for 3 h. The reaction was stopped by adding H_2_O (60 mL) at 0°C. The pH value was adjusted to 9 causing the precipitation of compound **8a**. The precipitate was filtered, washed with cold H_2_O (3 × 15 mL) and dried. Purification was managed by automated flash chromatography (Biotage® Sfär S C18 D; CH_3_CN/H_2_O), obtaining 143 mg of the aimed crude compound **8a** with a purity of 86%. A total of 55 mg were further purified by HPLC following the described method and product **8a** was obtained as a white solid after lyophilisation (13 mg, 0.04 mmol, 9%). *R*_*t*_ = 31–32 min (method C18). dp ≥ 265°C. Purity (HPLC *B*): > 99% (*R*_*t*_ = 5.3 min). Exact mass (ESI): m/z = calcd. for C_14_H_19_N_10_O_2_ [M + H^+^] 359.1687, found 359.1687. ^1^H NMR (600 MHz, DMSO‐*d*_6_): δ (ppm) = 10.53 (s, 1H, O*H*/N*H*_guanine_), 8.49 (s, 1H, N*H*_amide_), 7.72 (s, 1H, 2‐C*H*_pyrimidine_), 7.69 (s, 1H, 8‐C*H*_guanine_), 6.42 (s, 2H, N*H*_2‐guanine_), 5.81 (s, 4H, N*H*_2‐pyrimidine_), 3.94 (t, *J* = 7.1 Hz, 2H, 5‐C*H*_2‐pentyl_), 2.34 (t, *J* = 7.5 Hz, 2H, 2‐C*H*_2‐pentyl_), 1.77 (p, *J* = 7.2 Hz, 2H, 4‐C*H*_2‐pentyl_), 1.53 (p, *J* = 7.7 Hz, 2H, 3‐C*H*_2‐pentyl_). ^13^C NMR (151 MHz, DMSO‐*d*_6_): δ (ppm) = 171.6 (*C*=O_amide_), 159.6 (2 C, *C*−4,6_pyrimidine_), 155.3 (*C*−2_pyrimidine_), 151.1 (*C*−4_guanine_), 137.5 (*C*−8_guanine_), 116.6 (*C*−5_guanine_), 95.5 (*C*−5_pyrimidine_), 42.6 (*C*−5_pentyl_), 34.5 (*C*−2_pentyl_), 29.4 (*C*−4_pentyl_), 21.7 (*C*−3_pentyl_). FTIR (neat): $\tilde{v}$ (cm^−1^) = 3314 (N─H), 2727 (C─H), 1697 (C=O), 1620 (C=N).

#### 4.1.8 Synthesis of 6‐(2‐Amino‐6‐Hydroxy‐9*H*‐Purin‐9‐yl)‐*N*‐(4,6‐Diaminopyrimidin‐5‐yl)Hexanamide (**8b**)

The ester **7** (500 mg, 1.6 mmol, 1.0 eq.) was dissolved in eaton's reagent at 80°C under N_2_ and mixture was stirred for 5 min at 80°C. Pyrimidine‐4,5,6‐triamine (241 mg, 1.9 mmol, 1.2 eq.) was added to the solution and stirred for 3 h. The reaction was stopped by adjusting the pH value to 9 using aqueros NH_4_OH (~25%) causing precipitation. The precipitate was filtered, washed with cold H_2_O (3 × 15 mL) and dried. Purification was managed by automated flash chromatography (Biotage® Sfär S C18 D; CH_3_CN/H_2_O), obtaining 204 mg. A total of 67 mg of the crude product were further purified by semi preperative HPLC and product **8b** was obtained as a white solid after lyophilization (31 mg, 0.09 mmol, 15%). *R*_*t*_ = 33–36 min (method C18). dp ≥ 230°C. Purity (HPLC *B*): 96% (*R*_*t*_ = 6.2 min). Exact mass (ESI): m/z = calcd. for C_15_H_21_N_10_O_2_ [M + H^+^] 373.1843, found 373.1847. ^1^H NMR (600 MHz, DMSO‐*d*_6_): δ (ppm) = 10.54 (s, 1H, O*H*/N*H*_guanine_), 8.55 (s, 1H, N*H*_amide_), 7.80 (s, 1H, C*H*_pyrimidine_), 7.69 (s, 1H, 8‐C*H*_guanine_), 6.46 (s, 2H, N*H*_2‐guanine_), 6.08 (s, 4H, 4,6‐N*H*_2‐pyrimidine_), 3.93 (t, *J* = 7.2 Hz, 2H, 6‐C*H*_2‐hexyl_), 2.30 (m, 2H, 2‐C*H*_2‐hexyl_), 1.74 (p, *J* = 7.5 Hz, 2H, 5‐C*H*_2‐hexyl_), 1.59 (p, *J* = 7.7 Hz, 2H, 3‐C*H*_2‐hexyl_), 1.26 (m, 2H, 4‐C*H*_2‐hexyl_). ^13^C NMR (151 MHz, DMSO‐*d*_6_): δ (ppm) = 171.9 (*C*=O_amide_), 159.0 (2 C, *C*−4,6_pyrimidine_), 156.9 (*C*−6_guanine_), 154.1 (*C*−2_pyrimidine_), 153.5 (*C*−2_guanine_), 151.1 (*C*−4_guanine_), 137.5 (*C*−8_guanine_), 116.6 (*C*−5_guanine_), 95.3 (*C*−5_pyrimidine_), 42.6 (*C*−6_hexyl_), 34.9 (*C*−2_hexyl_), 29.4 (*C*−5_hexyl_), 25.9 (*C*−4_hexyl_), 24.1 (*C*−3_hexyl_). FTIR (neat): $\tilde{v}$ (cm^−1^) = 3318, 3194, 3148 (O─H/N─H), 2932, 2847 (C─H), 1636, 1585 (C=N).

#### 4.1.9 Synthesis of 5‐(2‐Amino‐6‐Hydroxy‐9*H*‐Purin‐9‐yl)‐*N*‐(4‐Aminopyrimidin‐5‐yl)Pentanamide (**8c**)

The ester **7** (200 mg, 0.7 mmol, 1 eq.) was dissolved in eaton's reagent at 80°C. The mixture was stirred for 5 min and pyrimidine‐4,5‐diamine (98 mg, 0.9 mmol, 1.2 eq.) was added. After stirring at 80°C for 2 h, the reaction was stopped by cooling and adjusting the pH value to 9 using aqueous NH_4_OH (~25%). The solvent was removed under reduced pressure. Residue was purified by automated flash chromatography (Biotage® Sfär S C18 D; CH_3_CN/H_2_O) twise and further purified via semi preperative HPLC. After lyophilisation product **8c** was obtained as a white solid (24 mg, 0.07 mmol, 9%). *R*_*t*_ = 32–35 min (method C18). dp ≥ 295°C. Purity (HPLC *B*): 98% (*R*_*t*_ = 7.6 min). Exact mass (ESI): m/z = calcd. for C_14_H_18_N_9_O_2_ [M + H^+^] 344.1578, found 344.1579. ^1^H NMR (600 MHz, DMSO‐*d*_6_): δ (ppm) = 10.52 (s, 1H, O*H*/N*H*_guanine_), 9.08 (s, 1H, N*H*_amide_), 8.21 (s, 1H, 6‐C*H*_pyrimidine_), 8.19 (s, 1H, 2‐C*H*_pyrimidine_), 7.69 (s, 1H, 8‐C*H*_guanine_), 5.69 (s, 2H, N*H*_2‐pyrimidine_), 6.42 (s, 2H, N*H*_2‐guanine_), 3.95 (t, *J* = 7.1 Hz, 2H, 5‐C*H*_2‐pentyl_), 2.35 (t, *J* = 7.5 Hz, 2H, 2‐C*H*_2‐pentyl_), 1.76 (p, *J* = 7.2 Hz, 2H, 4‐C*H*_2‐pentyl_), 1.54 (p, *J* = 7.7 Hz, 2H, 3‐C*H*_2‐pentyl_). ^13^C NMR (151 MHz, DMSO‐*d*_6_): δ (ppm) = 171.6 (*C*=O_amide_), 157.9 and 156.8 (*C*−2_guanine_ and *C*−6_purine_), 157.8 (*C*−4_pyrimidine_), 154.7 (*C*−2_pyrimidine_), 151.1 (*C*−4_guanine_), 149.7 (*C*−6_pyrimidine_), 137.5 (*C*−8_guanine_), 116.6 (*C*−5_guanine_), 116.6 (*C*−5_pyrimidine_), 42.5 (*C*−5_pentyl_), 35.0 (*C*−2_pentyl_), 29.1 (*C*−4_pentyl_), 22.0 (*C*−3_pentyl_). FTIR (neat): $\tilde{v}$ (cm^−1^) = 3175 (O─H/N─H), 3021, 2947, 2870 (C─H), 1667 (C=O), 1528 (C=N).

#### 4.1.10 Synthesis of 2‐Amino‐9‐[4‐(6‐Amino‐9*H*‐Purin‐8‐yl)Butyl]−9*H*‐Purin‐6‐ol (**9a**)

Compound **8a** (350 mg, 1.0 mmol, 1.0 eq.) was dissolved in aqueous NaOH solution (2 M, 30 mL) and solution was refluxed for 7 h. The reaction was stopped by cooling the mixture to 0°C and adjusting the pH value to 9 using aqueous concentrated HCl. The forming precipitate was filtered, washed with cold H_2_O (3 × 30 mL) and dried. Crude product was purified by automated flash chromatography (Biotage® Sfär S C18 D; CH_3_CN/H_2_O) followed by semi preperative HPLC. Compound **9a** was obtained as a white solid after lyophilisation (29 mg, 0.09 mmol, 8%). *R*_*t*_ = 37–40 min (method C18). dp ≥ 245°C. Purity (HPLC *B*): 97% (*R*_*t*_ = 9.5 min). Exact mass (ESI): m/z = calcd. for C_14_H_17_N_10_O [M + H^+^] 341.1581, found 341.1586. ^1^H NMR (600 MHz, DMSO‐*d*_6_): δ (ppm) = 8.03 (s, 1H, 2‐C*H*_adenine_), 7.69 (s, 1H, 8‐C*H*_guanine_), 6.91 (s, 2H, N*H*_2‐adenine_), 6.43 (s, 2H, N*H*_2‐guanine_), 3.96 (t, *J* = 7.0 Hz, 2H, 4‐C*H*_2‐butyl_), 2.78 (t, *J* = 7.5 Hz, 2H, 1‐C*H*_2‐butyl_), 1.77 (m, 2H, 3‐C*H*_2‐butyl_), 1.68 (m, 2H, 2‐C*H*_2‐butyl_). N*H*_adenine_ and N*H*/O*H*_guanine_ were not visible. ^13^C NMR (151 MHz, DMSO‐*d*_6_): δ (ppm) = 154.4 (*C*−4_adenine_), 151.7 (*C*−2_adenine_), 151.4 (*C*−8_adenine_), 151.2 (*C*−4_guanine_), 151.0 (*C*−4_adenine_), 137.5 (*C*−8_guanine_), 118.5 (*C*−5_adenine_), 116.6 (*C*−5_guanine_), 42.3 (*C*−4_butyl_), 28.1 (*C*−1_butyl_), 29.1 (*C*−3_butyl_), 24.6 (*C*−2_butyl_). FTIR (neat): $\tilde{v}$ (cm^−1^) = 3298, 3125 3102 (O─H/N─H), 2920, 2735 (C─H), 1643 (C=O), 1601 (C=N).

#### 4.1.11 General Procedure A for the Synthesis of Compounds **9b**–**9q**

The ester **6** or ester **7** was dissolved in eaton's reagent (25 mL per 100 mg) at 80°C, forming a clear solution. Di‐/triamine (1.2 eq.) was then added and the mixture was stirred at 80°C for 3 h. The reaction stopped by cooling to 0°C and adjusting the pH value to > 11 using aqueous NaOH (10 M). The solution was then refluxed overnight until it turned lighter in colour. The solution was cooled to 0°C, and pH was adjusted to 9 using aqueous concentrated HCl. After 10 min, the formed precipitate was filtered, and the residue was washed with H_2_O. The crude product was purified by automated flash chromatography (Biotage® Sfär S C18 D; CH_3_CN/H_2_O) followed by semi preperative HPLC. Fractions containing the product were parted, and the solvent was removed by reduced pressure and lyophilization.

2‐Amino‐9‐[5‐(6‐amino‐9*H*‐purin‐8‐yl)pentyl]−9*H*‐purin‐6‐ol (**9b**): Compound **9b** was prepared following the general procedure **A**: ester **7** (100 mg, 0.32 mmol, 1.0 eq.) and pyrimidine‐4,5,6‐triamine (48 mg, 0.38 mmol, 1.2 eq.) were used. The product **9b** was obtained as a white solid after purification by semi preparative HPLC (32 mg, 0.09 mmol, 28%). *R*_*t*_ = 41–43 min (method C18). dp ≥ 205°C. Purity (HPLC *B*): 99% (*R*_*t*_ = 10.2 min). Exact mass (ESI): m/z = calcd. for C_15_H_19_N_10_O [M + H^+^] 355.1738, found 355.1752. ^1^H NMR (600 MHz, DMSO‐*d*_6_): δ (ppm) = 8.04 (s, 1H, C*H*_adenine_), 7.67 (s, 1H, 8‐C*H*_guanine_), 6.89 (s, 2H, N*H*_2‐adenine_), 6.44 (s, 2H, N*H*_2‐guanine_), 3.92 (t, *J* = 7.2 Hz, 2H, 5‐C*H*_2‐pentyl_), 2.74 (t, *J* = 7.5 Hz, 2H, 1‐C*H*_2‐pentyl_), 1.75 (m, 4H, 2,4‐C*H*_2‐pentyl_), 1.29 (m, 2H, 3‐C*H*_2‐pentyl_). N*H*_adenine_ and N*H*/O*H*_guanine_ were not visible. ^13^C NMR (151 MHz, DMSO‐*d*_6_): δ (ppm) = 157.0 (*C*−6_guanine_), 153.6 (*C*−2_guanine_), 152.0 (*C*−8_adenine_), 151.7 (*C*−2_adenine_), 151.1 (*C*−4_guanine_), 137.4 (*C*−8_guanine_), 116.6 (*C*−5_guanine_), 42.4 (*C*−5_pentyl_), 28.5 (*C*−1_pentyl_), 29.2 (*C*−4_pentyl_), 26.9 (*C*−2_pentyl_) 225.6 (*C*−3_pentyl_). *C*−4_adenine_, *C*−5_adenine_, and *C*−6_adenine_ were not visible. FTIR (neat): $\tilde{v}$ (cm^−1^) = 3113 (N─H), 2943, 2743, (C─H), 1640 (C=O), 1589 (C─N) 1300 (C─O).

9‐[4‐(3*H*‐Imidazo[4,5‐*c*]pyridin‐2‐yl)butyl]−2‐amino‐9*H*‐purin‐6‐ol (**9c**): Compound **9c** was prepared following the general procedure **A**: Ester **6** (200 mg, 0.74 mmol, 1.0 eq.) and pyridine‐3,4‐diamine (97 mg, 0.89 mmol, 1.2 eq.) were used. The product **9c** was obtained as a light beige solid without the use of HPLC purification (101 mg, 0.31 mmol, 42%). dp ≥ 275°C. Purity (HPLC *B*): 96% (*R*_*t*_ = 5.6 min). Exact mass (ESI): m/z = calcd. for C_15_H_17_N_8_O [M + H^+^] 325.1520, found 325.1530. ^1^H NMR (600 MHz, DMSO‐*d*_6_): δ (ppm) = 8.77 (d, *J* = 1.0 Hz, 1*H*, 2‐C*H*_pyridine_), 8.21 (d, *J* = 5.4 Hz, 1H, 6‐C*H*_pyridine_), 7.68 (s, 1H, 8‐C*H*_guanine_), 7.45 (dd, *J* = 5.6/1.2 Hz, 1H, 5‐C*H*_pyridine_), 6.46 (s, 2H, N*H*_2‐guanine_), 3.97 (t, *J* = 6.9 Hz, 2H, 4‐C*H*_2‐butyl_), 2.88 (t, *J* = 7.4 Hz, 2H, 1‐C*H*_2‐butyl_), 1.81 (m, 2H, 3‐C*H*_2‐butyl_), 1.73 (m, 2H, 2‐C*H*_2‐butyl_). N*H*_imidazole_ and N*H*/O*H*_guanine_ were not visible. ^13^C NMR (151 MHz, DMSO‐*d*_6_): δ (ppm) = 157.4 (*C*−2_imidazole_), 157.3 (*C*−6_guanine_), 153.77 (*C*−2_guanine_), 151.2 (*C*−4_guanine_), 140.7 (*C*−6_pyridine_), 138.3 (*C*−2_pyridine_), 137.4 (*C*−8_guanine_), 116.6 (*C*−5_guanine_), 108.5 (*C*−5_pyridine_), 42.3 (*C*−4_butyl_), 29.1 (*C*−3_butyl_), 28.0 (*C*−1_butyl_), 24.4 (*C*−2_butyl_). *C*−3_pyridine_, and *C*−4_pyridine_ were not visible. FTIR (neat): $\tilde{v}$ (cm^−1^) = 3175, 2943, 2859, 2712 (C─H/N─H), 1624 (C=O), 1605 (C=N).

9‐[5‐(3*H*‐Imidazo[4,5‐*c*]pyridin‐2‐yl)pentyl]−2‐amino‐9*H*‐purin‐6‐ol (**9d**): Compound **9d** was prepared following the general procedure **A**: ester **7** (200 mg, 0.64 mmol, 1.0 eq.) and pyridine‐3,4‐diamine (84 mg, 0.77 mmol, 1.2 eq.) were used. The product was obtained as a white solid without HPLC purification (119 mg, 0.35 mmol, 55%). dp ≥ 270°C. Purity (HPLC *B*): 99% (*R*_*t*_ = 6.2 min). Exact mass (ESI): m/z = calcd. for C_16_H_19_N_8_O [M + H^+^] 339.1676, found 339.1684. ^1^H NMR (600 MHz, DMSO‐*d*_6_): δ (ppm) = 12.58 (s, 1H, N*H*_imidazole_), 10.51 (s, 1H, N*H*/O*H*_guanine_), 8.79 (s, 1*H*, 2‐C*H*_pyridine_), 8.23 (d, *J* = 5.4 Hz, 1H, 6‐C*H*_pyridine_), 7.67 (s, 1H, 8‐C*H*_guanine_), 7.44 (s, 1H, 5‐C*H*_pyridine_), 6.42 (s, 2H, N*H*_2‐guanine_), 3.92 (t, *J* = 7.2 Hz, 2H, 5‐C*H*_2‐pentyl_), 2.84 (t, *J* = 7.5 Hz, 2H, 1‐C*H*_2‐pentyl_), 1.81 (m, 2H, 2‐C*H*_2‐pentyl_), 1.76 (m, 2H, 4‐C*H*_2‐pentyl_), 1.31 (m, 2H, 3‐C*H*_2‐pentyl_). ^13^C NMR (151 MHz, DMSO‐*d*_6_): δ (ppm) = 156.8 (*C*−2_imidazole_), 156.8 (*C*−6_guanine_), 153.5 (*C*−2_guanine_), 151.1 (*C*−4_guanine_), 140.9 (*C*−6_pyridine_), 140.6 (*C*−2_pyridine_), 137.4 (*C*−8_guanine_), 116.6 (*C*−5_guanine_), 106.4 (*C*−5_pyridine_), 42.4 (*C*−5_pentyl_), 29.2 (*C*−4_pentyl_), 28.3 (*C*−1_pentyl_), 26.7 (*C*−2_pentyl_), 25.6 (*C*−3_pentyl_). *C*−3_pyridine_, and *C*−4_pyridine_ were not visible. FTIR (neat): $\tilde{v}$ (cm^−1^) = 3310, 3140 (O─H/N─H), 2932, 2866 (C─H), 1597 (C = N).

9‐[4‐(3*H*‐Imidazo[4,5‐*b*]pyridin‐2‐yl)butyl]−2‐amino‐9*H*‐purin‐6‐ol (**9e**): Compound **9e** was prepared following the general procedure **A**: ester **6** (200 mg, 0.74 mmol, 1.0 eq.) and pyridine‐2,3‐diamine (97 mg, 0.89 mmol, 1.2 eq.) were used. The produced was obtained as a light beige solid without the use of HPLC purification (7 mg, 0.02 mmol, 3%). dp ≥ 270°C. Purity (HPLC *B*): 96% (*R*_*t*_ = 10.1 min). Exact mass (ESI): m/z = calcd. for C_15_H_17_N_8_O [M + H^+^] 325.1520, found 325.1536. ^1^H NMR (600 MHz, DMSO‐*d*_6_): δ (ppm) = 8.23 (dd, *J* = 4.8/1.5 Hz, 1*H*, 6‐C*H*_pyridine_), 7.85 (d, *J* = 7.3 Hz, 1H, 4‐C*H*_pyridine_), 7.69 (s, 1H, 8‐C*H*_guanine_), 7.14 (dd, *J* = 7.9/4.8 Hz, 1H, 5‐C*H*_pyridine_), 6.44 (s, 2H, N*H*_2‐guanine_), 3.98 (t, *J* = 6.9 Hz, 2H, 4‐C*H*_2‐butyl_), 2.86 (t, *J* = 7.4 Hz, 2H, 1‐C*H*_2‐butyl_), 1.81 (m, 2H, 3‐C*H*_2‐butyl_), 1.74 (m, 2H, 2‐C*H*_2‐butyl_). N*H*_imidazole_ and N*H*/O*H*_guanine_ were not visible. ^13^C NMR (151 MHz, DMSO‐*d*_6_): δ (ppm) = 157.0 (*C*−6_guanine_), 156.8 (*C*−2_imidazole_), 153.59 (*C*−2_guanine_), 151.2 (*C*−4_guanine_), 142.6 (*C*−6_pyridine_), 137.4 (*C*−8_guanine_), 117.2 (*C*−5_pyridine_), 116.6 (*C*−5_guanine_), 42.3 (*C*−4_butyl_), 29.1 (*C*−3_butyl_), 28.1 (*C*−1_butyl_), 24. (*C*−2_butyl_). *C*−2_pyridine_ and *C*−3_pyridine_ were not visible. FTIR (neat): $\tilde{v}$ (cm^−1^) = 3464, 3313, 3163 (O─H/N─H), 3024, 2932, 2727 (C─H), 1701 (C=O), 1601 (C=N).

9‐[5‐(3*H*‐Imidazo[4,5‐*b*]pyridin‐2‐yl)pentyl]−2‐amino‐9*H*‐purin‐6‐ol (**9f**): Compound **9f** was prepared following the general procedure **A**: ester **7** (200 mg, 0.6 mmol, 1 eq.) and pyridine‐2,3‐triamine (84 mg, 0.77 mmol, 1.2 eq.) were used. Crude Product was purified by semi‐preparative HPLC. First following the described C18 method. Afterwards residue was purified again by semi preparative HPLC: solvent A = ACN, solvent B = H_2_O (0–2 min 2 mL/min ‐> 5 mL/min 85% B, 2–22 min 5 mL/min 85% B, 22–52 min 5 mL/min 85% ‐> 80% B, 52–67 min 80% ‐> 85% B, 67–77 min 5 mL/min 85% B, 77–78 min 5 mL/min ‐> 0.1 mL/min 85% B). Product **9f** was obtained as a yellow solid (23 mg, 0.07 mmol, 11%). dp ≥ 210°C. Purity (HPLC *B*): 96% (*R*_*t*_ = 6.3 min, 10.8 min). (protonated N7 and N9 for adenine moiety separating at HPLC column). Exact mass (ESI): m/z = calcd. for C_16_H_19_N_8_O [M + H^+^] 339.1676, found 339.1687. ^1^H NMR (600 MHz, DMSO‐*d*_6_): δ (ppm) = 10.51 (s, 1H, O*H*_guanine_), 8.23 (m, 1H, 6‐C*H*_pyridine_), 7.86 (m, 1H, 4‐C*H*_pyridine_), 7.67 (s, 1H, 8‐C*H*_guanine_), 7.15 (dd, *J* = 7.9/4.7 Hz, 1H, 5‐C*H*_pyridine_), 6.43 (s, 2H, N*H*_2‐guanine_), 3.92 (t, *J* = 7.2 Hz, 2H, 5‐C*H*_2‐pentyl_), 2.82 (t, *J* = 7.6 Hz, 2H, 1‐C*H*_2‐pentyl_), 1.81 (m, 2H, 2‐C*H*_2‐pentyl_), 1.76 (m, 2H, 4‐C*H*_2‐pentyl_), 1.31 (m, 2H, 3‐C*H*_2‐pentyl_). N*H*_imidazole_ was not visible. ^13^C NMR (151 MHz, DMSO‐*d*_6_): δ (ppm) = 156.8 (*C*−6_guanine_), 156.7 (*C*−2_imidazole_), 153.5 (*C*−2_guanine_), 151.1 (*C*−4_guanine_), 142.3 (*C*−6_pyridine_), 137.4 (*C*−8_guanine_), 117.2 (*C*−5_pyridine_), 116.6 (*C*−5_guanine_), 42.5 (*C*−5_pentyl_), 29.1 (*C*−4_pentyl_) 28.6 (*C*−1_pentyl_), 26.7 (*C*−2_pentyl_), 25.6 (*C*−3_pentyl_). *C*−2_pyridine_, *C*−3_pyridine_, and *C‐*4_pyridine_ were not visible. FTIR (neat): $\tilde{v}$ (cm^−1^) = 3305, 3097 (O─H/N─H), 2939, 2862 (C─H), 1651 (C=O), 1597 (C=N).

9‐[4‐(9*H*‐Purin‐8‐yl)butyl]−2‐amino‐9*H*‐purin‐6‐ol (**9g**): Compound **9g** was prepared following the general procedure **A**: ester **6** (200 mg, 0.7 mmol, 1 eq.) and pyrimidine‐4,5‐diamine (98 mg, 0.9 mmol, 1.2 eq.) were used. Product **9g** was obtained as a white solid after purification by semi preparative HPLC (44 mg, 0.14 mmol, 18%). *R*_*t*_ = 37–39 min (method C18). dp > 300°C. Purity (HPLC *B*): 96% (*R*_*t*_ = 9.8 min). Exact mass (ESI): m/z = calcd. for C_14_H_16_N_9_O [M + H^+^] 326.1472, found 326.1463. ^1^H NMR (600 MHz, DMSO‐*d*_6_): δ (ppm) = 10.51 (s, 1H, O*H*_guanine_), 8.94 and 8.81 (*C*−2_purine_ and *C*−6_purine_), 7.69 (s, 1H, 8‐C*H*_guanine_), 6.40 (s, 2H, N*H*_2‐guanine_), 3.98 (t, *J* = 6.8 Hz, 2H, 4‐C*H*_2‐butyl_), 2.91 (t, *J* = 7.4 Hz, 2H, 1‐C*H*_2‐butyl_), 1.75 (m, 2H, 2‐C*H*_2‐butyl_), 1.81 (m, 2H, 3‐C*H*_2‐butyl_). N*H*_purine_ was not visible. ^13^C NMR (151 MHz, DMSO‐*d*_6_): δ (ppm) = 159.2 (*C*−8_purine_), 151.2 (*C*−4_guanine_), 137.5 (*C*−8_guanine_), 118.5 (*C*−5_purine_), 116.6 (*C*−5_guanine_), 42.3 (*C*−4_butyl_), 28.3 (*C*−1_butyl_), 24.1 (*C*−2_butyl_), 24.6 (*C*−2_butyl_). *C*−2_purine_, *C*−4_purine_, and *C*−6_purine_ were not visible. FTIR (neat): $\tilde{v}$ (cm^−1^) = 3124 (O─H/N─H), 2712, 2646 (C─H), 1686 (C=O), 1693 (C=N).

9‐[5‐(9*H*‐Purin‐8‐yl)pentyl]−2‐amino‐9*H*‐purin‐6‐ol (**9h**): Compound **9h** was prepared following the general procedure **A**: ester **7** (200 mg, 0.6 mmol, 1 eq.) and pyrimidine‐4,5‐diamine (85 mg, 0.8 mmol, 1.2 eq.) were used. The product **9h** was obtained as a white solid after purification by semi‐preparative HPLC (52 mg, 0.15 mmol, 24%). dp ≥ 259°C. Purity (HPLC *B*): 100% (*R*_*t*_ = 10.4 min). Exact mass (ESI): m/z = calcd. for C_16_H_19_N_9_O [M + H^+^] 340.1629, found 340.1632. ^1^H NMR (600 MHz, DMSO‐*d*_6_): δ (ppm) = 13.24 (s, 1H, N*H*_purine_), 12.94 (s, 0.45H, 7‐N*H*_purine_), 10.50 (s, 1H, O*H*/N*H*_*guanine*_), 8.85 (s, 1H, 9‐N*H*_purine_), 8.79 (s, 1H, 2‐C*H*_purine_), 8.95 (s, 1H, 6‐C*H*_purine_), 7.67 (s, 1H, 8‐C*H*_guanine_), 6.41 (s, 2H, N*H*_2‐guanine_), 3.92 (t, *J* = 7.2 Hz, 2H, 5‐C*H*_2‐pentyl_), 2.87 (m, 2H, 1‐C*H*_2‐pentyl_), 1.82 (p, *J* = 7.7 Hz, 2H, 2‐C*H*_2‐pentyl_), 1.76 (p, *J* = 7.5 Hz, 2H, 4‐C*H*_2‐pentyl_), 1.32 (m, 2H, 3‐C*H*_2‐pentyl_). The NMR shows a mixture of N9 and N7 protonated purine in a ratio of 0.45–1.00. This explains the singulets at δ 12.94 and δ 8.85. ^13^C NMR (151 MHz, DMSO‐*d*_6_): δ (ppm) = 158.4 (*C*−8_purine_), 156.8 (*C*−6_guanine_), 153.4 (*C*−2_guanine_), 153.1 (*C*−4_purine_), 151.5 (*C*−2_purine_), 151.1 (*C*−4_guanine_), 145.6 (*C*−6_purine_), 137.4 (*C*−8_guanine_), 133.9 (*C*−5_purine_), 116.6 (*C*−5_guanine_), 42.4 (*C*−5_pentyl_), 29.2 (*C*−4_pentyl_), 28.6 (*C*−1_pentyl_), 26.4 (*C*−2_pentyl_), 25.5 (*C*−3_pentyl_). The ^13^C NMR also displays a ratio of N7 and N9 protonated derivates of the purine. FTIR (neat): $\tilde{v}$ (cm^−1^) = 3429, 3294, 3179 (O─H/N─H), 2924, 2859 (C─H), 1705 (C=O), 1608 (C=N).

2‐Amino‐9‐[4‐(7‐amino‐3*H*‐imidazo[4,5‐*c*]pyridin‐2‐yl)butyl]−9*H*‐purin‐6‐ol (**9i**): Compound **9i** was prepared following the general procedure **A**: ester **6** (200 mg, 0.7 mmol, 1 eq.) and pyridine‐3,4,5‐triamine (110 mg, 0.9 mmol, 1.2 eq.) were used. The product was obtained as a slightly beige solid without the use of HPLC purification (45 mg, 0.35 mmol, 18%). dp ≥ 195°C. Purity (HPLC *B*): 98% (*R*_*t*_ = 5.4 min). Exact mass (ESI): m/z = calcd. for C_15_H_18_N_9_O [M + H^+^] 340.1629, found 340.1637. ^1^H NMR (600 MHz, DMSO‐*d*_6_): δ (ppm) = 8.05 (s, 1H, 2‐C*H*_pyridine_), 7.68 (s, 1H, 8‐C*H*_guanine_), 7.62 (s, 1H, 6‐C*H*_pyridine_), 6.46 (s, 2H, N*H*_2‐guanine_), 5.18 (s, 2H, N*H*_2‐pyridine_), 3.97 (t, *J* = 6.9 Hz, 2H, 4‐C*H*_2‐butyl_), 2.84 (t, *J* = 7.5 Hz, 2H, 1‐C*H*_2‐butyl_), 1.80 (p, *J* = 7.0 Hz, 2H, 3‐C*H*_2‐butyl_), 1.71 (p, *J* = 7.7 Hz, 2H, 2‐C*H*_2‐butyl_). N*H*_imidazole_ and N*H*/O*H*_guanine_ were not visible. ^13^C NMR (151 MHz, DMSO‐*d*_6_): δ (ppm) = 157.3 (*C*−6_guanine_), 155.0 (*C*−2_imidazole_), 153.8 (*C*−2_guanine_), 151.2 (*C*−4_guanine_), 137.4 (*C*−8_guanine_), 125.5 (*C*−6_pyridine_), 116.6 (*C*−5_guanine_), 42.3 (*C*−4_butyl_), 29.1 (*C*−3_butyl_), 28.0 (*C*−1_butyl_), 24.7 (*C*−2_butyl_). *C*−2_pyridine_, *C*−3_pyridine_, *C*−4_pyridine_, and *C*−5_pyridine_ were not visible. FTIR (neat): $\tilde{v}$ (cm^−1^) = 3329, 3159 (N─H), 2935, 2855, 2700 (C─H), 1682 (C=O), 1605 (C─N).

2‐Amino‐9‐[5‐(7‐amino‐3*H*‐imidazo[4,5‐*c*]pyridin‐2‐yl)pentyl]−9*H*‐purin‐6‐ol (**9j**): Compound **9j** was prepared following the general procedure **A**: ester **7** (200 mg, 0.6 mmol, 1 eq.) and pyridine‐3,4,5‐triamine (96 mg, 0.8 mmol, 1.2 eq.) were used. The product **9j** was obtained as a slightly beige solid without the use of HPLC purification (44 mg, 0.12 mmol, 19%). dp ≥ 180°C. Purity (HPLC *B*): 96% (*R*_*t*_ = 5.7 min). Exact mass (ESI): m/z = calcd. for C_16_H_20_N_9_O [M + H^+^] 354.1785, found 354.1798. ^1^H NMR (600 MHz, DMSO‐*d*_6_): δ (ppm) = 7.96 (s, 1H, 2‐C*H*_pyridine_), 7.57 (s, 1H, 8‐C*H*_guanine_), 7.52 (s, 1H, 6‐C*H*_pyridine_), 6.36 (s, 2H, N*H*_2‐guanine_), 5.08 (s, 2H, N*H*_2‐pyridine_), 3.82 (t, *J* = 7.2 Hz, 2H, 5‐C*H*_2‐pentyl_), 2.70 (t, *J* = 7.5 Hz, 2H, 1‐C*H*_2‐pentyl_), 1.68 (m, 4H, 2,4‐C*H*_2‐pentyl_), 1.20 (p, *J* = 7.8 Hz, 2H, 3‐C*H*_2‐pentyl_). N*H*_imidazole_ and N*H*/O*H*_guanine_ were not visible. ^13^C NMR (151 MHz, DMSO‐*d*_6_): δ (ppm) = 157.1 (*C*−6_guanine_), 155.0 (*C*−2_imidazole_), 153.7 (*C*−2_guanine_), 151.2 (*C*−4_guanine_), 137.4 (*C*−8_guanine_), 125.8 (*C*−6_pyridine_), 116.6 (*C*−5_guanine_), 42.2 (*C*−5_pentyl_), 29.0 (*C*−4_pentyl_), 28.3 (*C*−2_pentyl_), 27.0 (*C*−1_pentyl_), 25.6 (*C*−3_pentyl_). *C*−2_pyridine_, *C*−3_pyridine_, *C*−4_pyridine_, and *C*−5_pyridine_ were not visible. FTIR (neat): $\tilde{v}$ (cm^−1^) = 3321, 3186 (O─H/N─H), 3048, 2928, 2635 (C─H), 1713 (C=O), 1620, 1609 (C=N).

2‐Amino‐9‐[4‐(2‐chloro‐9*H*‐purin‐8‐yl)butyl]−9*H*‐purin‐6‐ol (**9k**): Compound **9k** was prepared following the general procedure **A**: ester **6** (200 mg, 0.7 mmol, 1 eq.) and 6‐chloropyrimidine‐4,5‐diamine (129 mg, 0.9 mmol, 1.2 eq.) were used. The product **9k** was obtained as a white solid after purification by semi preparative HPLC (54 mg, 0.15 mmol, 20%). dp ≥ 250°C. Purity (HPLC *B*): 100% (*R*_*t*_ = 9.8 min). Exact mass (ESI): m/z = calcd. for C_14_H_15_^35^ClN_9_O [M + H^+^] 360.1083, found 360.1091. ^1^H NMR (600 MHz, DMSO‐*d*_6_): δ (ppm) = 10.50 (s, 1H, O*H*/N*H*_guanine_), 8.49 (s, 1H, 2‐C*H*_purine_), 7.69 (s, 1H, 8‐C*H*_guanine_), 6.42 (s, 2H, N*H*_2‐guanine_), 3.97 (t, *J* = 6.9 Hz, 2H, 4‐C*H*_2‐butyl_), 2.87 (t, *J* = 7.4 Hz, 2H, 1‐C*H*_2‐butyl_), 1.81 (m, 2H, 3‐C*H*_2‐butyl_), 1.74 (m, 2H, 2‐C*H*_2‐butyl_). N*H*_purine_ was not visible. ^13^C NMR (151 MHz, DMSO‐*d*_6_): δ (ppm) = 162.5 (*C*−8_purine_), 157.2 and 144.6 (*C*−4_purine_ and *C*−6_purine_), 156.8 (*C*−6_guanine_), 153.5 (*C*−2_guanine_), 151.1 (*C*−4_guanine_), 149.5 (*C*−2_purine_), 137.5 (*C*−8_guanine_), 116.6 (*C*−5_guanine_), 42.3 (*C*−4_butyl_), 29.3 (*C*−3_butyl_), 29.1 (*C*−1_butyl_), 24.5 (*C*−2_butyl_). *C*−5_purine_ was not visible. FTIR (neat): $\tilde{v}$ (cm^−1^) = 3345, 3186 (O─H)/N─H, 2916, 2820., 2743 (C─H), 1690 (C=O), 1601 (C=N).

2‐Amino‐9‐[5‐(2‐chloro‐9*H*‐purin‐8‐yl)pentyl]−9*H*‐purin‐6‐ol (**9l**): Compound **9l** was prepared following the general procedure **A**: ester **7** (200 mg, 0.6 mmol, 1 eq.) and 6‐chloropyrimidine‐4,5‐diamine (111 mg, 0.8 mmol, 1.2 eq.) were used. The product **9l** was obtained as a white solid after purification by semi preparative HPLC. (54 mg, 0.15 mmol, 24%). *R*_t_ = 34–39 min (method C18). dp ≥ 257°C. Purity (HPLC *B*): 97% (*R*_*t*_ = 10.6 min). Exact mass (ESI): m/z = calcd. for C_15_H_17_^35^ClN_9_O [M + H^+^] 374.1239, found 374.1232. ^1^H NMR (600 MHz, DMSO‐*d*_6_): δ (ppm) = 13.62 (s, 1H, N*H*_purine_), 10.49 (s, 1H, O*H*/N*H*_guanine_), 8.64 (s, 1H, 2‐C*H*_purine_), 7.68 (s, 1H, 8‐C*H*_guanine_), 6.41 (s, 2H, N*H*_2‐guanine_), 3.93 (t, *J* = 7.2 Hz, 2H, 5‐C*H*_2‐pentyl_), 2.88 (t, *J* = 7.6 Hz, 2H, 1‐C*H*_2‐pentyl_), 1.82 (p, *J* = 7.7 MHz, 2H, 2‐C*H*_2‐pentyl_), 1.77 (p, *J* = 7.4 MHz, 2H, 4‐C*H*_2‐pentyl_), 1.32 (m, 2H, 3‐C*H*_2‐pentyl_). ^13^C NMR (151 MHz, DMSO‐*d*_6_): δ (ppm) = 157.3 (*C*−8_purine_), 156.8 (*C*−6_guanine_), 153.4 (*C*−2_guanine_), 151.1 (*C*−4_guanine_), 150.8 (*C*−2_purine_), 137.4 (*C*−8_guanine_), 116.6 (*C*−5_guanine_), 42.4 (*C*−5_pentyl_), 29.1 (*C*−4_pentyl_), 28.7 (*C*−1_pentyl_), 26.4 (*C*−2_pentyl_), 25.5 (*C*−3_pentyl_). *C*−4_purine_, *C*−5_purine_, and *C*−6_purine_ were not visible. FTIR (neat): $\tilde{v}$ (cm^−1^) = 3375 (O─H/N─H), 2909, 2731, (C─H), 1678 (C=O).

8‐[4‐(2‐Amino‐6‐hydroxy‐9*H*‐purin‐9‐yl)butyl]−9*H*‐purin‐2‐ol (**9m**): Compound **9m** was prepared following the general procedure **A**: ester **6** (200 mg, 0.7 mmol, 1 eq.) and 6‐chloropyrimidine‐4,5‐diamine (129 mg, 0.9 mmol, 1.2 eq.) were used. The product **9m** was obtained as a white solid after purification by semi preparative HPLC (22 mg, 0.06 mmol, 9%). *R*_*t*_ = 30–34 min (method C18). dp ≥ 292°C. Purity (HPLC *B*): 98% (*R*_*t*_ = 8.3 min, 9.8 min, protonated N7 and N9 for purine moiety separating at HPLC column). Exact mass (ESI): m/z = calcd. for C_14_H_16_N_9_O_2_ [M + H^+^] 342.1421 found 342.1409. ^1^H NMR (600 MHz, DMSO‐*d*_6_): δ (ppm) = 7.90 (s, 1H, 2‐C*H*_purine_), 7.68 (s, 1H, 8‐C*H*_guanine_), 6.43 (s, 2H, N*H*_2‐guanine_), 3.95 (t, *J* = 7.0 Hz, 2H, 4‐C*H*_2‐butyl_), 2.75 (t, *J* = 7.4 Hz, 2H, 1‐C*H*_2‐butyl_), 1.76 (m, 2H, 3‐C*H*_2‐butyl_), 1.67 (m, 2H, 2‐C*H*_2‐butyl_). N*H*_purine_ and N*H*/O*H*_guanine_ were not visible. ^13^C NMR (151 MHz, DMSO‐*d*_6_): δ (ppm) = 156.8 (*C*−6_guanine_), 153.5 (*C*−2_guanine_), 151.1 (*C*−4_guanine_), 144.2 (*C*−2_purine_), 137.4 (*C*−8_guanine_), 116.6 (*C*−5_guanine_), 42.2 (*C*−4_butyl_), 29.0 (*C*−3_butyl_), 27.8 (*C*−1_butyl_), 24.5 (*C*−2_butyl_). *C*−4_purine_, *C*−5_purine_, *C*−6_purine_, and *C*−8_purine_ were not visible. FTIR (neat): $\tilde{v}$ (cm^−1^) = 3422 (O─H/N─H), 2943, 2808., (C─H), 1713 (C=O), 1624 (C=N).

8‐[5‐(2‐Amino‐6‐hydroxy‐9*H*‐purin‐9‐yl)pentyl]−9*H*‐purin‐2‐ol (**9n**): Compound **9n** was prepared following the general procedure **A**: ester **7** (200 mg, 0.6 mmol, 1 eq.) and 6‐chloropyrimidine‐4,5‐diamine (111 mg, 0.8 mmol, 1.2 eq.) were used. The crude product was purified by semi‐preparative HPLC. First following the described C18 method. Afterwards residue was purified again by semi‐preparative HPLC: solvent A = ACN, solvent B = H_2_O (0–2 min 2 mL/min ‐> 5 mL/min 90% B, 2–30 min 5 mL/min 90% B, 30–31 min 5 mL/min ‐> 0.1 mL/min 90% B). Product **9n** was obtained as a white solid (17 mg, 0.05 mmol, 7%). *R*_*t*_ = 46–47 min (method C18). dp ≥ 230°C. Purity (HPLC *B*): 100% (*R*_*t*_ = 8.8 min, 10.6 min, protonated N7 and N9 purine derivatives separated at the HPLC column) Exact mass (ESI): m/z = calcd. for C_15_H_18_N_9_O_2_ [M + H^+^] 356.1578 found 356.1581. ^1^H NMR (600 MHz, DMSO‐*d*_6_): δ (ppm) = 10.50 (s, 1H, O*H*/N*H*_guanine/purine_), 7.91 (s, 1H, 2‐C*H*_purine_), 7.67 (s, 1H, 8‐C*H*_guanine_), 6.43 (s, 2H, N*H*_2‐guanine_), 3.91 (t, *J* = 7.3 Hz, 2H, 5‐C*H*_2‐pentyl_), 2.71 (t, *J* = 7.4 Hz, 2H, 1‐C*H*_2‐pentyl_), 1.74 (p, *J* = 7.5 MHz, 4H, 2,4‐C*H*_2‐pentyl_), 1.27 (m, 2H, 3‐C*H*_2‐pentyl_). N*H*_purine_ was not visible. ^13^C NMR (151 MHz, DMSO‐*d*_6_): δ (ppm) = 156.8 (*C*−6_guanine_), 154.5 (*C*−8_purine_), 153.5 (*C*−2_guanine_), 151.1 (*C*−4_guanine_), 144.0 (*C*−2_purine_), 137.4 (*C*−8_guanine_), 116.6 (*C*−5_guanine_), 42.4 (*C*−5_pentyl_), 29.2 (*C*−4_pentyl_), 28.2 (*C*−1_‐pentyl_), 26.8 (*C*−2_pentyl_), 25.5 (*C*−3_pentyl_). *C*−4_purine_, *C*−5_purine_, and *C*−6_purine_ were not visible. FTIR (neat): $\tilde{v}$ (cm^−1^) = 3445, 3321, 3106 (O─H/N─H), 2924, 2862, 2770 (C─H), 1701 (C=O), 1643. 1535 (C=N).

2‐Amino‐9‐[4‐(5‐chloro‐3*H*‐imidazo[4,5‐*b*]pyridin‐2‐yl)butyl]−9*H*‐purin‐6‐ol (**9o**): Compound **9o** was prepared following the general procedure **A**: ester **6** (150 mg, 0.6 mmol, 1 eq.) and 6‐chloropyridine‐2,3‐diamine (96 mg, 0.7 mmol, 1.2 eq.) were used. Product **9o** was obtained as a beige solid without the use of HPLC purification (58 mg, 0.16 mmol, 29%). dp ≥ 240°C. Purity (HPLC *B*): 100% (*R*_*t*_ = 11.2 min). Exact mass (ESI): m/z = calcd. for C_15_H_16_^35^ClN_8_O [M + H^+^] 359.1130, found 359.1143. ^1^H NMR (600 MHz, DMSO‐*d*_6_): δ (ppm) = 7.91 (d, *J* = 8.2 Hz, 1H, 4‐C*H*_pyridine_), 7.69 (s, 1H, 8‐C*H*_guanine_), 7.20 (d, *J* = 8.3 Hz, 1H, 5‐C*H*_pyridine_), 6.41 (s, 2H, N*H*_2‐guanine_), 3.97 (t, *J* = 6.9 Hz, 2H, 4‐C*H*_2‐butyl_), 2.87 (t, *J* = 7.4 Hz, 2H, 1‐C*H*_2‐butyl_), 1.80 (m, 2H, 3‐C*H*_2‐butyl_), 1.72 (m, 2H, 2‐C*H*_2‐butyl_). N*H*_imidazole_ and N*H*/O*H*_guanine_ were not visible. ^13^C NMR (151 MHz, DMSO‐*d*_6_): δ (ppm) = 158.1 (C‐2_imidazole_), 156.8 (C‐6_guanine_), 153.5 (C‐2_guanine_), 151.2 (C‐4_guanine_), 142.8 (C‐6_pyridine_), 137.4 (*C*−8_guanine_), 116.9 (*C*−5_guanine_), 116.6 (*C*−5_pyridine_), 42.3 (*C*−4_butyl_), 29.0 (*C*−3_butyl_), 28.3 (*C*−1_butyl_), 24.3 (*C*−2_butyl_). *C*−2_pyridine_, *C*−3_pyridine_, and *C*−4_pyridine_ were not visible. FTIR (neat): $\tilde{v}$ (cm^−1^) = 3337, 3105 (O─H/N─H), 2940, 2770 (C─H), 1685, 1643 (C=O), 1609 (C=N).

2‐Amino‐9‐[5‐(5‐chloro‐3*H*‐imidazo[4,5‐*b*]pyridin‐2‐yl)pentyl]−9*H*‐purin‐6‐ol (**9p**): Compound **9p** was prepared following the general procedure **A**: ester **7** (150 mg, 0.5 mmol, 1 eq.) and 6‐chloropyridine‐2,3‐diamine (83 mg, 0.6 mmol, 1.2 eq.) were used. The product **9p** was obtained as a slightly beige solid without the use of HPLC purification (40 mg, 0.11 mmol, 22%). dp ≥ 250°C. Purity (HPLC *B*): > 99% (*R*_*t*_ = 11.8 min). Exact mass (ESI): m/z = calcd. for C_16_H_18_^35^ClN_8_O [M + H^+^] 373.1287, found 373.1300. ^1^H NMR (600 MHz, DMSO‐*d*_6_): δ (ppm) = 7.91 (s, 1H, 4‐C*H*_pyridine_), 7.67 (s, 1H, 8‐C*H*_guanine_), 7.21 (s, 1H, 5‐C*H*_pyridine_), 6.43 (s, 2H, N*H*_2‐guanine_), 3.92 (t, *J* = 7.3 Hz, 2H, 5‐C*H*_2‐pentyl_), 2.83 (t, *J* = 7.5 Hz, 2H, 1‐C*H*_2‐pentyl_), 1.80 (m, 2H, 2‐C*H*_2‐pentyl_), 1.76 (m, 2H, 4‐C*H*_2‐pentyl_), 1.31 (m, 2H, 3‐C*H*_2‐pentyl_). N*H*_imidazole_ and N*H*/O*H*_guanine_ were not visible. ^13^C NMR (151 MHz, DMSO‐*d*_6_): δ (ppm) = 159.0 (*C*−2_imidazole_), 156.9 (*C*−6_guanine_), 153.5 (*C*−2_guanine_), 151.1 (*C‐*4_guanine_), 142.7 (*C*−6_pyridine_), 137.4 (*C*−8_guanine_), 116.9 (*C*−5_pyridine_), 116.6 (*C*−5_guanine_), 42.4 (*C*−2_pentyl_), 29.2 (*C*−4_pentyl_), 28.7 (*C*−1_pentyl_), 26.6 (*C*–2_pentyl_), 25.6 (*C*–3_pentyl_). *C*−2_pyridine_, *C*−3_pyridine_, and *C*−4_pyridine_ were not visible. FTIR (neat): $\tilde{v}$ (cm^−1^) = 3476, 3341, 3171, 3125 (O─H/N─H), 3048, 2947, 2727 (C─H), 1685 (C=O), 1620 (C=N).

2‐Amino‐9‐[5‐(4‐amino‐1*H*‐imidazo[4,5‐*c*]pyridin‐2‐yl)pentyl]−9*H*‐purin‐6‐ol and 2‐amino‐9‐[5‐(7‐amino‐3*H*‐imidazo[4,5‐*b*]pyridin‐2‐yl)pentyl]−9*H*‐purin‐6‐ol (**9q**): Compound **9q** was prepared following the general procedure **A**: ester **7** (300 mg, 1.0 mmol, 1 eq.) and pyridine‐2,3,4‐triamine (143 mg, 1.2 mmol, 1.2 eq.) were used. The product was obtained as a slightly beige mixture of 4‐amino‐1H‐imidazo[4,5‐c]pyridin‐2‐yl and 7‐amino‐3H‐imidazo[4,5‐b]pyridin‐2‐yl derivatives, in a ratio of 0.69–0.31, respectively. Only a mixture could be purified as both regioisomers could not be separated by HPLC purification (60 mg, 0.17 mmol, 18%). C_16_H_19_N_9_O (353.39 g/mol) dp ≥ 172°C. Purity (HPLC *B*): 100% for mixture of both regioisomers (*R*_*t*_ = 5.8 min). Exact mass (ESI): m/z = calcd. for C_16_H_20_N_9_O [M + H^+^] 354.1785, found 354.1763. ^1^H NMR (600 MHz, DMSO‐*d*_6_): δ (ppm) = 12.17 (s, 1.45H, N*H*_imidazole_), 10.54 (s, 1.45H, O*H*/N*H*_guanine_), 7.69 (m, 1H, 6‐C*H*_pyridine_), 7.68 (s, 1.45H, 8‐C*H*_guanine_), 7.57 (d, *J* = 5.7 Hz, 1H, 6‐C*H*_pyridine_), 6.65 (s, 1H, 5‐C*H*_pyridine_),6.44 (s, 2.90H, N*H*_2‐guanine_), 6.26 (m, 1H, 5‐C*H*_pyridine_), 3.92 (m, 2.90H, 5‐C*H*_2‐pentyl_), 2.76 (t, *J* = 7.5 Hz, 2H, 1‐C*H*_2‐pentyl_), 2.72 (t, *J* = 7.0 Hz, 2H, 1‐C*H*_2‐pentyl_), 1.76 (m, 5.80H, 2,4‐C*H*_2‐pentyl_), 1.29 (m, 2.9H, 3‐C*H*_2‐pentyl_). ^13^C NMR (151 MHz, DMSO‐*d*_6_): δ (ppm) = 156.8 (1.45 C, *C*−6_guanine_), 153.5 (1.45 C, *C*−2_guanine_), 151.4 (*C*−4_pyridine_), 151.4 (*C*−2_imidazole_), 151.1 (1.45 C, *C*−4_guanine_), 151.1 (*C*−2_imidazole_), 143.5 (*C*−6_pyridine_), 139.5 (1.45 C, *C*−6_pyridine_), 139.2 (*C*−6_pyridine_), 138.3 (*C*−2_pyridine_), 137.4 (1.45 C, *C*−8_guanine_), 126.5 (*C*−3_pyridine_), 116.6 (1.45 C, *C*−5_guanine_), 101.78 (*C*−5_pyridine_), 97.8 (*C*−5_pyridine_), 42.4 (1.45 C, *C*−5_pentyl_), 29.2 (1.45 C, *C*−4_pentyl_) 28.6 (*C*−1_pentyl_), 28.3 (*C*−1_pentyl_), 27.2 (*C*−2_pentyl_), 27.1 (*C*−2_pentyl_), 25.6 (*C*−3_pentyl_). FTIR (neat): $\tilde{v}$ (cm^−1^) = 3321, 3174 (O─H/N─H), 3090, 2936, 2727 (C─H), 1716 (C=O).

#### 4.1.12 Synthesis of 6‐Chloro‐9‐(Hex‐5‐yn‐1‐yl)−9*H*‐Purin‐2‐Amine (12)

2‐Amino‐6‐chloropurine (**2**, 1 g, 5.90 mmol, 1 eq.) and K_2_CO_3_ (1.63 g, 11.80 mmol, 2 eq.) were suspended in dry DMF under N_2_ atmosphere and the mixture was stirred at 0°C for 2 h. 6‐Iodo‐1‐hexyne (1.6 mL, 12.14 mmol, 2.1 eq.) was added slowly to the reaction mixture, then the mixture was allowed to warm to room temperature and to stir for 1 day. The solvent was removed in vacuo, the residue was dissolved in CH_2_Cl_2_ (50 mL) and washed with H_2_O (20 mL) and brine (20 mL). The combined organic layers were dried over Na_2_SO_4_ and the solvent was removed in vacuo. The crude product was purified via flash chromatography (CH_3_OH/ethyl acetate = 2:98; Ø = 4 cm, *l* = 12 cm) and product **12** was obtained as a white solid (1.09 g, 4.38 mmol, 74%). mp = 106.2°C. Purity (HPLC *A*): > 99% (*R*_*t*_ = 7.9 min). Exact mass (ESI): m/z = calcd. for C_11_H_13_ClN_5_ [M + H^+^] 250.0859, found 250.0867. ^1^H NMR (600 MHz, DMSO‐*d*_6_): δ (ppm) = 8.13 (s, 1H, 8‐C*H*_purine_), 6.89 (s, 2H, N*H*_*2‐*purine_), 4.05 (t, *J* = 7.0 Hz, 2H, *1‐*C*H*_*2‐*hex‐5‐yn‐1‐yl_), 2.76 (t, *J* = 2.7 Hz, 1H, 6‐C*H*_hex‐5‐yn‐1‐yl_), 2.19 (td, *J* = 7.1, 2.7 Hz, 2H, 4*‐*C*H*_*2‐*hex‐5‐yn‐1‐yl_), 1.85 (p, *J* = 7.3 Hz, 2H, 2*‐*C*H*_*2‐*hex‐5‐yn‐1‐yl_), 1.40 (p, *J* = 7.1 Hz, 2H, 3‐C*H*_*2‐*hex‐5‐yn‐1‐yl_). ^13^C NMR (151 MHz, DMSO‐*d*_6_): δ (ppm) = 159.7 (*C*−6_purine_), 154.1 (*C*−4_purine_), 149.3 (*C*−2_purine_), 143.2 (*C*−8_purine_), 123.3 (*C*−5_purine_), 84.0 (*C*−5_hex‐5‐yn‐1‐yl_), 71.5 (*C*−6_hex‐5‐yn‐1‐yl_), 42.4 (*C*−1_hex‐5‐yn‐1‐yl_), 28.1 (*C*−2_hex‐5‐yn‐1‐yl_), 25.0 (*C*−3_hex‐5‐yn‐1‐yl_), 17.2 (*C*−4_hex‐5‐yn‐1‐yl_). FTIR (neat): $\tilde{v}$ (cm^−1^) = 3429, 3329 (N─H), 3210 (C─H_alkyne_), 2928 (C─H_aliphatic_), 1647, 1608 (C=C/C=N), 1558 (N─H_bend_), 1350, 1277 (C─N).

#### 4.1.13 Synthesis of 6‐Chloro‐9‐(Pent‐4‐yn‐1‐yl)−9*H*‐Purin‐2‐Amine (**13**)

2‐Amino‐6‐chlropurine (**2**, 543 mg, 3.20 mmol, 1 eq.) and K_2_CO_3_ (885.8 g, 6.41 mmol, 2 eq.) were suspended in dry DMF under N_2_ atmosphere and the mixture was stirred at 0°C for 2 h. 5‐Bromo‐1‐pentyne (0.45 mL, 4.17 mmol, 1.3 eq.) was added slowly to the reaction mixture, then the mixture was allowed to warm to room temperature and stirred for 1 day. The solvent was removed in vacuo, the residue was dissolved in CH_2_Cl_2_ (20 mL), washed with H_2_O (10 mL) and brine (10 mL). The combined organic layers were dried over Na_2_SO_4_ and the solvent was removed in vacuo. The crude product was purified via flash chromatography (CH_3_OH/ethyl acetate = 4:96; Ø = 3 cm, *l* = 12 cm) and the product **13** was obtained as a white solid (593.30 mg, 2.52 mmol, 79%). mp = 143.6°C. Purity (HPLC *A*): 100% (*R*_*t*_ = 6.5 min). Exact mass (APCI): m/z = calcd. for C_10_H_11_ClN_5_ [M + H^+^] 236.0703, found 235.0673. ^1^H NMR (600 MHz, DMSO‐*d*_6_): δ (ppm) = 8.11 (s, 1H, 8‐C*H*_purine_), 6.90 (s, 2H, N*H*_*2‐*purine_), 4.13–4.08 (m, 2H, 1*‐*C*H*_*2‐*pent‐4‐yn‐1‐yl_), 2.85–2.81 (m, 1H, 5*‐*C*H*_*‐*pent‐4‐yn‐1‐yl_), 2.20 (td, *J* = 7.1, 2.7 Hz, 2H, 3*‐*C*H*_*2‐*pent‐4‐yn‐1‐yl_), 1.98 (p, *J* = 7.1 Hz, 2H, 2*‐*C*H*_*2*‐ pent‐4‐yn‐1‐yl_). ^13^C NMR (151 MHz, DMSO‐*d*_6_): δ (ppm) = 159.7 (*C*−6_purine_), 154.1 (*C*−4_purine_), 149.3 (*C*−2_purine_), 143.2 (*C*−8_purine_), 123.4 (*C*−5_purine_), 83.0 (*C*−4_pent‐4‐yn‐1‐yl_), 72.0 (*C*−5_pent‐4‐yn‐1‐yl_), 42.3 (*C*−1_pent‐4‐yn‐1‐yl_), 27.5 (*C*−2_pent‐4‐yn‐1‐yl_), 15.2 (*C*−3_pent‐4‐yn‐1‐yl_). FTIR (neat): $\tilde{v}$ (cm^−1^) = 3345, 3302 (N─H), 3190 (C─H_alkyne_), 2978 (C─H_aliphatic_), 1637, 1612 (C=C/C=N), 1562 (N─H_bend_), 1354, 1285 (C─N).

#### 4.1.14 Synthesis of 8‐Azidoadenosine (**15**)

8‐Bromoadenosine (**14**, 300 mg, 0.86 mmol, 1 eq.) was reacted with NaN_3_ (211 mg, 3.25 mmol, 3.78 eq.) in DMF (10 mL) and the mixture was stirred at 60°C for 18 h. The solvent was evaporated in vacuo, the resulting product was crystallized from H_2_O/CH_3_OH mixture, filtered and the collected crystals were washed with H_2_O, giving product **15** as a beige solid (131 mg, 0.42 mmol, 50%). mp = 189.3°C. Purity (HPLC *C*): 89% (*R*_*t*_ = 4.0 min), with 11% of 8‐Bromoadenosine. Exact mass (APCI): m/z = calcd. for C_10_H_13_N_8_O_4_ [M + H^+^] 309.1060, found 309.0974, 346.0163 for 8‐Bromoadenosine. ^1^H NMR (600 MHz, DMSO‐*d*_6_): δ (ppm) = 8.08 (s, 1H, 2‐C*H*_adenosine_), 7.31 (s, 2H, N*H*_*2‐*adenosine_), 5.64 (d, *J* = 6.6 Hz, 1H, 1′‐C*H*_adenosine_), 5.42–5.37 (m, 2H, 5′‐O*H*_adenosine_ and 2′‐O*H*_adenosine_), 5.16 (s, 1H, 3′‐O*H*_adenosine_), 4.90 (t, *J* = 6.0 Hz, 1H, 2′‐C*H*_adenosine_), 4.16 (s, 1H, 3′‐C*H*_adenosine_), 3.92 (td, *J* = 4.2, 2.6 Hz, 1H, 4′‐C*H*_adenosine_), 3.65 (dt, *J* = 12.1, 4.1 Hz, 1H, 5′‐C*H*_adenosine_), 3.51 (ddd, *J* = 12.3, 8.3, 4.3 Hz, 1H, 5′‐C*H*_adenosine_). ^13^C NMR (151 MHz, DMSO‐*d*_6_): δ (ppm) = 154.5 (*C*−6_adenosine_), 151.6 (*C*−2_adenosine_), 149.3 (*C*−4_adenosine_), 144.4 (*C*−8_adenosine_), 117.1 (*C*−5_adenosine_), 87.6 (*C*−1′_adenosine_), 86.3 (*C*−4′_adenosine_), 71.3 (*C*−2′_adenosine_), 70.8 (*C*−3′_adenosine_), 62.1 (*C*−5′_adenosine_). FTIR (neat): $\tilde{v}$ (cm^−1^) = 3429 (O─H/N─H), 2978 (C─H_aliphatic_), 2141 (N=N=N), 1659, 1605 (C=C/C=N), 1574 (N─H_bend_), 1331 (C─N), 1130, 1084, 1045 (C─O).

#### 4.1.15 Synthesis of ((3a*R*,4*R*,6*R*,6a*R*)−6‐(6‐Amino‐8‐Bromo‐9*H*‐Purin‐9‐yl)−2,2‐Dimethyltetrahydrofuro[3,4‐*d*][1,3]Dioxol‐4‐yl)Methanol (**16**)

8‐Bromoadenosine (**14**, 2 g, 5.784 mmol, 1 eq.) was dissolved in acetone (50 mL). 2,2‐Dimethoxypropane (10.6 mL, 86.66 mmol, 15 eq.) and *p*‐TsOH (1.65 g, 8.67 mmol, 1.5 eq.) were added and the reaction mixture was stirred at room temperature for 1 day. The mixture was diluted with brine (15 mL), saturated NaHCO_3_ was added, and the mixture was extracted with ethyl acetate (2 × 45 mL). The combined organic layers were dried over Na_2_SO_4_, filtered and the solvent was removed under reduced pressure. The residue was purified by flash chromatography (CH_3_OH/ethyl acetate = 4:96; Ø = 5 cm, *l* = 15 cm) and product **16** was obtained as a white solid (1.52 g, 3.96 mmol, 68%). Purity (HPLC *A*): 97% (*R*_*t*_ = 7.8 min). Exact mass (APCI): m/z = calcd. for C_13_H_17_BrN_5_O_4_ [M + H^+^] 386.0464, found 386.0464. ^1^H NMR (600 MHz, DMSO‐*d*_6_): δ (ppm) = 8.15 (s, 1H, 2‐C*H*_adenosine_), 7.54 (s, 1H, N*H*_*2‐*adenosine_), 6.02 (d, *J* = 2.7 Hz, 1H, 1′‐C*H*_adenosine_), 5.66 (dd, *J* = 6.2, 2.7 Hz, 1H, 2′‐C*H*_adenosine_), 5.11 (dd, *J* = 6.5, 5.3 Hz, 1H, 5′‐O*H*_adenosine_), 5.03 (dd, *J* = 6.2, 3.0 Hz, 1H, 3′‐C*H*_adenosine_), 4.16 (td, *J* = 5.9, 3.0 Hz, 1H, 4′‐C*H*_adenosine_), 3.52 (dt, *J* = 11.4, 5.6 Hz, 1H, 5′‐C*H*_adenosine_), 3.43 (dt, *J* = 11.8, 6.1 Hz, 1H, 5′‐C*H*_adenosine_), 1.55 (s, 3H, C(C*H*_3_)_2_), 1.33 (s, 3H, C(C*H*_3_)_2_). ^13^C NMR (151 MHz, DMSO‐*d*_6_): δ (ppm) = 154.9 (*C*−6_adenosine_), 152.7 (*C*−2_adenosine_), 149.6 (*C*−4_adenosine_), 126.2 (*C*−8_adenosine_), 119.1 (*C*−5_adenosine_), 113.1 (*C*(CH_3_)_2_), 90.8 (*C*−1′_adenosine_), 87.0 (*C*−4′_adenosine_), 81.8 (*C*−2′_adenosine_), 81.5 (*C*−3′_adenosine_), 61.3 (*C*−5′_adenosine_), 27.0 (C(*C*H_3_)_2_), 25.1 (C(*C*H_3_)_2_). FTIR (neat): $\tilde{v}$ (cm^−1^) = 3318, 3197 (O─H/N─H), 1643, 1604 (C=C/C=N), 1574 (N─H_bend_), 1373 (C─N), 1157, 1103, 1064 (C─O), 656 (C‐Br).

#### 4.1.16 Synthesis of (2*R*,3*R*,4*S*,5*R*)−2‐(6‐Amino‐9*H*‐Purin‐9‐yl)−5‐(Azidomethyl)tetrahydrofuran‐3,4‐diol (18) or 5′‐Azido‐5′‐Deoxyadenosine (**18**)

Adenosine (**17**, 1 g, 3.74 mmol, 1 eq.) was suspended in CH_3_CN (8 mL) and cooled in ice bath to 0°C. Thionyl chloride (1.4 mL, 19.3 mmol, 5 eq.) was added, followed by the addition of pyridine (0.61 mL, 7.48 mmol, 2 eq) and the reaction mixture was stirred at 0°C for 4 h. The mixture was allowed to warm to room temperature and stirred for 18 h. The resulting precipitate was filtered and dissolved in H_2_O/CH_3_OH (5:1, 30 mL) and aqueous NH_3_ (25%, 2 mL) was added. The mixture was stirred at room temperature for 30 min and the solvent was removed in vacuo to provide 5′‐chloro‐5′‐deoxyadenosine which was used directly without further purification. 5′‐Chloro‐5′‐deoxyadenosine was dissolved in DMF (8 mL) and NaN_3_ (1.2 g, 18.6 mmol, 4.94 eq.) was added. The reaction mixture was heated at 80°C for 5 h and then filtered to remove the excess of NaN_3_. The solvent was evaporated in vacuo, the residue was purified by flash chromatography (CH_3_OH/CH_2_Cl_2_ = 20:80; Ø = 3 cm, *l* = 15 cm) and product **18** was obtained as a white solid (436 mg, 0.007 mmol, 40% as two isomeric forms with a 1:0.4 ratio). mp = 82.1°C. Purity (HPLC *A*): 94% (*R*_*t*_ = 3.1 min). Exact mass (APCI): m/z = calcd. for C_10_H_13_N_8_O_3_ [M + H^+^] 293.1111, found 293.1060. ^1^H NMR (600 MHz, DMSO‐*d*_6_): δ (ppm) = δ 8.35 (s, 1H, 8‐C*H*_adenosine‐major_), 8.34 (s, 0.38H, 8‐C*H*_adenosine‐minor_), 8.16 (s, 1H, 2‐C*H*_adenosine‐major_), 8.157 (s, 0.34H, 2‐C*H*_adenosine‐minor_), 7.29 (s, 2H, N*H*_2*‐*adenosine‐major_), 7.31 (s, 0.74H, N*H*_2*‐*adenosine‐minor_), 5.94–5.91 (m, 1.46H, 1'‐C*H*_adenosine‐major_, 1′‐C*H*_adenosine‐minor_), 5.59 (d, *J* = 6.0 Hz, 0.44H, 2′‐O*H*_adenosine‐minor_), 5.57 (d, *J* = 5.9 Hz, 1H, 2′‐O*H*_adenosine‐major_), 5.45 (d, *J* = 5.2 Hz, 0.41H, 1H, 3′‐O*H*_adenosine‐minor_), 5.37 (d, *J* = 5.1 Hz, 1H, 3′‐O*H*_adenosine‐major_), 4.78–4.72 (m, 1.53H, 2′‐C*H*_adenosine‐major_, 2′‐C*H*_adenosine‐minor_), 4.24–4.22 (m, 0.43H, 3′‐C*H*_adenosine‐minor_), 4.20 (q, *J* = 4.9 Hz, 1H, 3′‐C*H*_adenosine‐major_), 4.10–4.07 (m, 0.43H, 4′‐C*H*_adenosine‐minor_), 4.06–4.03 (m, 1H, 4′‐C*H*_adenosine‐major_), 3.95 (dd, *J* = 11.6, 5.1 Hz, 0.45H, 5′‐C*H*_adenosine‐minor_), 3.84 (dd, *J* = 11.6, 6.4 Hz, 0.44H, 5′‐C*H*_adenosine‐minor_), 3.68 (dd, *J* = 13.2, 7.1 Hz, 1H, 5′‐C*H*_adenosine‐major_), 3.55 (dd, *J* = 13.1, 3.7 Hz, 1H, 5′‐C*H*_adenosine‐major_). ^13^C NMR (151 MHz, DMSO‐*d*_6_): δ (ppm) = 156.1 (*C*−6_adenosine‐major,_ *C*−6_adenosine‐minor_), 152.7 (*C*−2_adenosine‐major_, *C*−2_adenosine‐minor_), 149.4 (*C*−4_adenosine‐major_, *C*−4_adenosine‐minor_), 139.9 (*C*−8_adenosine‐major_), 139.7 (*C*−8_adenosine‐minor_), 119.2 (*C*−5_adenosine‐major_), 119.1 (*C*−5_adenosine‐minor_), 87.7 (*C*−1′_adenosine‐major_), 87.4 (*C*−1′_adenosine‐minor_), 83.7 (*C*−4′_adenosine‐minor_), 82.9 (*C*−4′_adenosine‐major_), 72.7 (*C*−2′_adenosine‐major_), 72.6 (*C*−2′_adenosine‐minor_), 71.3 (*C*−3′_adenosine‐minor_), 70.9 (*C*−3′_adenosine‐major_), 51.70 (*C*−5′_adenosine‐major_), 44.81 (*C*−5′_adenosine‐minor_). FTIR (neat): $\tilde{v}$ (cm^−1^) = 3642, 3318, 3140 (O─H/N─H), 2978 (C─H), 2106 (N=N=N), 1670, 1601 (C=C/C=N), 1574 (N─H_bend_), 1130, 1099 (C─O).

#### 4.1.17 Synthesis of (2*R*,3*R*,4*S*,5*R*)−2‐(6‐Amino‐8‐{4‐[4‐(2‐Amino‐6‐Chloro‐9*H*‐Purin‐9‐yl)Butyl]−1*H*−1,2,3‐Triazol‐1‐yl}−9*H*‐Purin‐9‐yl)−5‐(Hydroxymethyl)tetrahydrofuran‐3,4‐diol (**19**)

Alkyne derivative **12** (50 mg, 0.20 mmol, 1 eq.) and 8‐azidoadenosine (**15**, 62 mg, 0.20 mmol, 1eq.) were dissolved in dry EtOH (1.5 mL) under N_2_ atmosphere. Copper(II) acetate (7 mg, 0.04 mmol, 0.2 eq.) and sodium ascorbate (20 mg, 0.10 mmol, 0.5 eq.) were added to the reaction mixture, and the mixture was stirred at 50°C for 2 days. The mixture was filtered through Celite, the solvent was evaporated, and the residue was purified by flash chromatography (Biotage® Sfär S C18 D; CH_3_CN/H_2_O = 10:90 → 100:0, 12 mL/min, 12 g) followed by further purification by flash chromatography (CH_3_OH/CH_2_Cl_2_ = 20:80; Ø = 1 cm, *l* = 15 cm). The product **19** was obtained as a pale‐yellow solid (6 mg, 0.01 mmol, 5%). *R*_*f*_ = 0.64 (20/80 CH_3_OH/CH_2_Cl_2_). mp = 153.9°C. Purity (HPLC *C*): 98% (*R*_*t*_ = 11.4 min). Exact mass (LC‐MS): m/z = calcd. for C_21_H_25_^35^ClN_13_O_4_ [M + H^+^] 558.1836, found 558.1794. ^1^H NMR (600 MHz, DMSO‐*d*_6_): δ (ppm) = 8.49 (s, 1H, 5‐C*H*_triazole_), 8.25 (s, 1H, 2‐C*H*_adenosine_), 8.17 (s, 1H, 8‐C*H*_purine_), 7.70 (s, 2H, N*H*_2‐adenosine_), 6.90 (s, 2H, N*H*_2‐purine_), 5.65 (d, *J* = 6.6 Hz, 1H, 1′‐C*H*_adenosine_), 5.45 (dd, *J* = 8.6, 4.0 Hz, 1H, 5′‐O*H*_adenosine_), 5.39 (d, *J* = 6.2 Hz, 1H, 2′‐O*H*_adenosine_), 5.16 (d, *J* = 4.5 Hz, 1H, 3′‐O*H*_adenosine_), 5.00 (q, *J* = 6.1 Hz, 1H, 2′‐C*H*_adenosine_), 4.14–4.09 (m, 3H, 4‐C*H*_*2‐*butyl_, 3′‐C*H*_adenosine_), 3.92 (q, *J* = 3.7 Hz, 1H, 4′‐C*H*_adenosine_), 3.65 (dt, *J* = 12.1, 4.0 Hz, 1H, 5′‐C*H*_adenosine_), 3.49 (ddd, *J* = 12.4, 8.6, 4.2 Hz, 1H, 5′‐C*H*_adenosine_), 2.82 (t, *J* = 7.6 Hz, 2H, 1‐C*H*_*2‐*butyl_), 1.88 (p, *J* = 7.2 Hz, 2H, 3‐C*H*_*2‐*butyl_), 1.66 (p, *J* = 7.6 Hz, 2H, 2‐C*H*_*2‐*butyl_). ^13^C NMR (151 MHz, DMSO‐*d*_6_): δ (ppm) = 159.7 (*C*−6_purine_), 156.4 (*C*−6_adenosine_), 154.1 (*C*−4_purine_), 153.5 (*C*−2_adenosine_), 149.3 (*C*−2_purine_), 149.1 (*C*−4_adenosine_), 147.2 (C‐4_triazole_), 143.3 (*C*−8_purine_), 139.3 (*C*−8_adenosine_), 124.9 (*C*−5_triazole_), 123.3 (*C*−5_purine_), 116.9 (*C*−5_adenosine_), 89.2 (*C*−1′_adenosine_), 86.7 (*C*−4′_adenosine_), 71.4 (*C*−2′_adenosine_), 70.7 (*C*−3′_adenosine_), 62.0 (*C*−5′_adenosine_), 42.7 (*C*−4_butyl_), 28.5 (*C*−3_butyl_), 25.8 (*C*−2_butyl_), 24.1 (*C*−1_butyl_). FTIR (neat): $\tilde{v}$ (cm^−1^) = 3318, 3186 (O─H/N─H), 2978, 2924 (C─H), 1609 (C=C/C=N), 1562 (N─H_bend_), 1080, 1038 (C─O).

#### 4.1.18 Synthesis of (2*R*,3*R*,4*S*,5*R*)−2‐(6‐Amino‐8‐{4‐[3‐(2‐Amino‐6‐Chloro‐9*H*‐Purin‐9‐yl)Propyl]−1*H*−1,2,3‐Triazol‐1‐yl}−9*H*‐Purin‐9‐yl)−5‐(Hydroxymethyl)tetrahydrofuran‐3,4‐diol (**20**)

Alkyne derivative **13** (50 mg, 0.21 mmol, 1 eq.) and 8‐azidoadenosine (**15**, 65 mg, 0.21 mmol, 1eq.) were dissolved in dry EtOH (2 mL) under N_2_ atmosphere. Copper(II) acetate (8 mg, 0.04 mmol, 0.2 eq.) and sodium ascorbate (21 mg, 0.10 mmol, 0.5 eq.) were added to the reaction mixture, and the mixture was stirred at 50°C for 2 days. The mixture was filtered through Celite, the solvent was evaporated, and the residue was purified by flash chromatography (CH_3_OH/CH_2_Cl_2_ = 20:80; Ø = 2 cm, *l* = 19 cm), obtaining product **20** as an off‐white solid (4 mg, 0.007 mmol, 3%). *R*_*f*_ = 0.59 (20/80 CH_3_OH/CH_2_Cl_2_). mp = 132.4°C. Purity (HPLC *C*): 98% (*R*_*t*_ = 10.5 min). Exact mass (ESI): m/z = calcd. for C_01_H_23_^35^ClN_13_O_4_ [M + H^+^] 544.1679, found 544.1710. ^1^H NMR (600 MHz, DMSO‐*d*_6_): δ (ppm) = 8.55 (d, *J* = 0.7 Hz, 1H, 5‐C*H*_*triazole*_), 8.26 (s, 1H, 2‐C*H*_adenosine_), 8.19 (s, 1H, 8‐C*H*_purine_), 7.71 (s, 2H, N*H*_2*‐*adenosine_), 6.91 (s, 2H, N*H*_2*‐*purine_), 5.64 (d, *J* = 6.6 Hz, 1H, 1′‐C*H*_adenosine_), 5.45 (dd, *J* = 8.6, 4.0 Hz, 1H, 5′‐O*H*_adenosine_), 5.39 (d, *J* = 6.2 Hz, 1H, 2′‐O*H*_adenosine_), 5.16 (d, *J* = 4.6 Hz, 1H, 3′‐O*H*_adenosine_), 5.00 (td, *J* = 6.4, 5.2 Hz, 1H, 2′‐C*H*_adenosine_), 4.17 (t, *J* = 7.1 Hz, 2H, 3‐C*H*_*2‐*propyl_), 4.12 (td, *J* = 4.8, 2.5 Hz, 1H, 3′‐C*H*_adenosine_), 3.94–3.90 (m, 1H, 4'‐C*H*_adenosine_), 3.65 (dt, *J* = 12.1, 4.0 Hz, 1H, 5′‐C*H*_adenosine_), 3.50 (ddd, *J* = 12.4, 8.6, 4.2 Hz, 1H, 5′‐C*H*_adenosine_), 2.79 (t, *J* = 7.6 Hz, 2H, 1‐C*H*_*2‐*propyl_), 2.24 (p, *J* = 7.4 Hz, 2H, 2‐C*H*_*2‐*propyl_). ^13^C NMR (151 MHz, DMSO‐*d*_6_): δ (ppm) = 159.8 (*C*−6_purine_), 156.4 (*C*−6_adenosine_), 154.1 (*C*−4_purine_), 153.5 (*C*−2_adenosine_), 149.4 (*C*−2_purine_), 149.1 (*C*−4_adenosine_), 146.4 (*C*−4_triazole_), 143.3 (*C*−8_purine_), 139.2 (*C*−8_adenosine_), 125.1 (*C*−5_triazole_), 123.4 (*C*−5_purine_), 116.9 (*C*−5_adenosine_), 89.2 (*C*−1′_adenosine_), 86.7 (*C*−4′_adenosine_), 71.5 (*C*−2′_adenosine_), 70.7 (*C*−3′_adenosine_), 62.0 (*C*−5′_adenosine_), 42.6 (*C*−3_propyl_), 28.4 (*C*−2_propyl_), 21.9 (*C*−1_propyl_). FTIR (neat): $\tilde{v}$ (cm^−1^) = 3318 (O─H/N─H), 2978, 2924 (C─H), 1609 (C=C/C=N), 1562 (N─H_bend_), 1076 (C─O).

#### 4.1.19 Synthesis of (2*R*,3*R*,4*S*,5*R*)−2‐{6‐Amino‐8‐[6‐(2‐Amino‐6‐Chloro‐9*H*‐Purin‐9‐yl)Hex‐1‐yn‐1‐yl)−9*H*‐Purin‐9‐yl)−5‐(Hydroxymethyl)tetrahydrofuran‐3,4‐diol (**21**)

Alkyne derivative **12** (500 mg, 2.00 mmol, 2 eq) and 8‐bromoadenosine derivative (**16**, 386.6 mg 1.00 mmol, 1 eq) were dissolved dry DMF (15 mL) under N_2_ atmosphere. CuI (38.1 mg, 0.20 mmol, 0.2 eq) and bis(triphenylphosphine) palladium dichloride (70.3 mg, 0.1 mmol, 0.1 eq) were added to the reaction mixture, followed by the addition of Et_3_N (0.7 mL, 5.00 mmol, 5 eq) and then the mixture was stirred at 80°C overnight. The solvent was removed in vacuo, the residue was dissolved in CH_3_OH (20 mL), filtered through Celite and the solvent was removed in vacuo. The residue was purified by flash chromatography (CH_3_OH/CH_2_Cl_2_ = 10:90; Ø = 4 cm, *l* = 15 cm) and the product was obtained as a yellow solid (304.6 mg, 0.55 mmol, 55%). Purity (HPLC *C*): 99% (*R*_*t*_ = 14.2 min). Exact mass (APCI): m/z = calcd. for C_24_H_28_^35^ClN_10_O_4_ [M + H^+^] 555.1978, found 555.1988. ^1^H NMR (600 MHz, DMSO‐*d*_6_): δ (ppm) = 8.164 and 8.161 (s, 2H, 2‐C*H*_adenosine_ and 8‐C*H*_purine_), 7.54 (s, 2H, N*H*_2*‐*adenosine_), 6.89 (s, 2H, N*H*_2*‐*purine_), 6.09 (d, *J* = 3.2 Hz, 1H, 1′‐C*H*_adenosine_), 5.50 (dd, *J* = 6.2, 3.1 Hz, 1H, 2′‐C*H*_adenosine_), 5.24 (dd, *J* = 6.9, 5.0 Hz, 1H, 5′‐O*H*_adenosine_), 4.99 (dd, *J* = 6.2, 2.9 Hz, 1H, 3′‐C*H*_adenosine_), 4.16 (td, *J* = 5.4, 2.9 Hz, 1H, 4′‐C*H*_adenosine_), 4.11 (t, *J* = 7.0 Hz, 2H, 6*‐*C*H*_*2‐* hex‐1‐yn‐1‐yl_), 3.57 (dt, *J* = 11.4, 5.2 Hz, 1H, 5′‐C*H*_adenosine_), 3.47 (ddd, *J* = 11.4, 6.9, 5.4 Hz, 1H, 5′‐C*H*_adenosine_), 2.66 (t, *J* = 7.1 Hz, 2H, 3*‐*C*H*_*2‐*hex‐1‐yn‐1‐yl_), 1.94 (dq, *J* = 9.6, 7.2 Hz, 2H, 2H, 5*‐*C*H*_*2‐*hex‐1‐yn‐1‐yl_), 1.57 (dq, *J* = 9.9, 7.2 Hz, 2H, 4*‐*C*H*_*2‐*hex‐1‐yn‐1‐yl_), 1.48 (s, 3H, C(C*H*_3_)_2_), 1.30 (s, 3H, C(C*H*_3_)_2_). ^13^C NMR (151 MHz, DMSO‐*d*_6_): δ (ppm) = 159.8 (*C*−6_purine_), 155.9 (*C*−6_adenosine_), 154.1 (*C*−4_purine_), 153.4 (*C*−2_adenosine_), 149.4 (*C*−2_purine_), 148.1 (*C*−4_adenosine_), 143.2 (*C*−8_purine_), 133.1 (*C*−8_adenosine_), 123.3 (*C*−5_purine_), 118.7 (*C*−5_adenosine_), 113.2 (*C*(CH_3_)_2_), 97.4 (*C*−2_hex‐1‐yn‐1‐yl_), 90.0 (*C*−1′_adenosine_), 86.6 (*C*−4′_adenosine_), 82.0 (*C*−2′_adenosine_), 81.6 (*C*−3′_adenosine_), 70.2 (*C*−1_hex‐1‐yn‐1‐yl_), 61.6 (*C*−5′_adenosine_), 42.3 (*C*−6_hex‐1‐yn‐1‐yl_), 28.3 (*C*−5_hex‐1‐yn‐1‐yl_), 27.1 (C(*C*H_3_)_2_), 25.2 (C(*C*H_3_)_2_), 24.6 (*C*−4_hex‐1‐yn‐1‐yl_), 18.0 (*C*−3_hex‐1‐yn‐1‐yl_). FTIR (neat): $\tilde{v}$ (cm^−1^) = 2978, 2935 (C─H), 1604 (C=C/C=N), 1562 (N─H_bend_), 1373 (C─N), 1153, 1080 (C─O).

To the protected nucleoside (60.3 mg, 0.11 mmol, 1 eq.), 7 N NH_3_ in CH_3_OH (1 mL) was added and the reaction mixture was stirred at 50°C in a sealed tube for 2 h. The solvent was evaporated under reduced pressure and the residue was dissolved in H_2_O/CH_3_OH (1:1, 3 mL) and Dowex resin (H^+^ form, 61 mg) was added. The mixture was stirred at 50°C for 5 h. Next a saturated solution of NaHCO_3_ was added at room temperature and the mixture was stirred for 30 min. The excess resin was removed by filtration and the filtrate was evaporated under reduced pressure. The residue was purified by flash chromatography (CH_3_OH/CH_2_Cl_2_ = 20:80; Ø = 1 cm, *l* = 20 cm), and product **21** was obtained as an off‐white solid (11.9 mg, 0.02 mmol, 21%). *R*_*f*_ = 0.42 (20/80 CH_3_OH/CH_2_Cl_2_). Purity (HPLC *C*): 100% (*R*_*t*_ = 11.3 min). Exact mass (ESI): m/z = calcd. for C_21_H_24_^35^ClN_10_O_4_ [M + H^+^] 515.1665, found 515.1679. ^1^H NMR (600 MHz, DMSO‐*d*_6_): δ (ppm) = 8.18 (s, 1H, 8‐C*H*_purine_), 8.13 (s, 1H, 2‐C*H*_adenosine_), 7.54 (s, 2H, N*H*_2*‐*adenosine_), 6.90 (s, 2H, N*H*_2*‐*purine_), 5.93 (d, *J* = 6.8 Hz, 1H, 1′‐C*H*_adenosine_), 5.56 (dd, *J* = 8.8, 3.8 Hz, 1H, 5′‐O*H*_adenosine_), 5.41 (d, *J* = 6.2 Hz, 1H, 2′‐O*H*_adenosine_), 5.19 (d, *J* = 4.3 Hz, 1H, 3′‐O*H*_adenosine_), 4.99 (td, *J* = 6.6, 5.2 Hz, 1H, 2′‐C*H*_adenosine_), 4.19 (td, *J* = 4.7, 2.3 Hz, 1H, 3′‐C*H*_adenosine_), 4.12 (t, *J* = 7.0 Hz, 2H, 6*‐*C*H*_*2‐*hex‐1‐yn‐1‐yl_), 3.97 (td, *J* = 3.9, 2.2 Hz, 1H, 4′‐C*H*_adenosine_), 3.67 (dt, *J* = 12.1, 3.9 Hz, 1H, 5'‐C*H*_adenosine_), 3.52 (ddd, *J* = 12.5, 8.8, 4.1 Hz, 1H, 5′‐C*H*_adenosine_), 2.64 (t, *J* = 7.0 Hz, 2H, 3*‐*C*H*_*2‐*hex‐1‐yn‐1‐yl_), 1.95 (p, *J* = 7.0 Hz, 2H, 5*‐*C*H*_*2‐*hex‐1‐yn‐1‐yl_), 1.56 (p, *J* = 7.2 Hz, 2H, 4*‐*C*H*_*2‐*hex‐1‐yn‐1‐yl_). ^13^C NMR (151 MHz, DMSO‐*d*_6_): δ (ppm) = 159.7 (*C*−6_purine_), 156.0 (*C*−6_adenosine_), 154.1 (*C*−4_purine_), 153.0 (*C*−2_adenosine_), 149.3 (*C*−2_purine_), 148.3 (*C*−4_adenosine_), 143.2 (*C*−8_purine_), 133.9 (*C*−8_adenosine_), 123.3 (*C*−5_purine_), 119.1 (*C*−5_adenosine_), 97.0 (*C*−2_hex‐1‐yn‐1‐yl_), 89.3 (*C*−1′_adenosine_), 86.6 (*C*−4′_adenosine_), 71.5 (*C*−2′_adenosine_), 71.0 (*C*−3_adenosine_), 70.5 (*C*−1_hex‐1‐yn‐1‐yl_), 62.2 (*C*−5′_adenosine_), 42.4 (*C*−6_hex‐1‐yn‐1‐yl_), 28.2 (*C*−5_hex‐1‐yn‐1‐yl_), 24.6 (*C*−4_hex‐1‐yn‐1‐yl_), 18.0 (*C*−3_hex‐1‐yn‐1‐yl_). FTIR (neat): $\tilde{v}$ (cm^−1^) = 3321 (O─H/N─H), 2928 (C─H), 2241 (C≡C), 1612 (C=C/C=N), 1562 (N─Hbend), 1396 (C─N), 1034 (C─O).

#### 4.1.20 Synthesis of (2*R*,3*R*,4*S*,5*R*)−2‐{6‐Amino‐8‐[5‐(2‐Amino‐6‐Chloro‐9*H*‐Purin‐9‐yl]Pent‐1‐yn‐1‐yl‐9*H*‐Purin‐9‐yl)−5‐(Hydroxymethyl)tetrahydrofuran‐3,4‐diol (**22**)

Alkyne derivative **13** (200 mg, 0.85 mmol, 2 eq.) and 8‐bromoadenosine derivative (**16**, 164 mg 0.42 mmol, 1 eq.) were dissolved in dry DMF (6 mL) under N_2_ atmosphere. CuI (16 mg, 0.09 mmol, 0.2 eq.) and bis(triphenylphosphine)palladium dichloride (30 mg, 0.04 mmol, 0.1 eq.) were added to the reaction mixture, followed by the addition of Et_3_N (0.3 mL, 2.12 mmol, 5 eq.), and then the mixture was stirred at 80°C overnight. The solvent was removed in vacuo, the residue was dissolved in CH_3_OH, filtered through Celite, and the solvent was removed in vacuo. The residue was purified by flash chromatography (CH_3_OH/CH_2_Cl_2_ = 10:90; Ø = 2 cm, *l* = 20 cm), and the product was obtained as a yellow solid (101 mg, 0.19 mmol, 44%). *R*_*f*_ = 0.53 (10/90 CH_3_OH/CH_2_Cl_2_). Purity (HPLC *C*): 98% (*R*_*t*_ = 13.6 min). Exact mass (ESI): m/z = calcd. for C_23_H_26_^35^ClN_10_O_4_ [M + H^+^] 541.1822, found 541.1842. ^1^H NMR (600 MHz, DMSO‐*d*_6_): δ (ppm) = 8.17 (s, 1H, 2‐C*H*_adenosine_), 8.14 (s, 1H, 8‐C*H*_purine_), 7.55 (s, 2H, N*H*_2*‐*adenosine_), 6.89 (s, 2H, N*H*_2*‐*purine_), 6.14 (d, *J* = 3.0 Hz, 1H, 1′‐C*H*_adenosine_), 5.52 (dd, *J* = 6.2, 3.0 Hz, 1H, 2′‐C*H*_adenosine_), 5.24 (dd, *J* = 6.8, 5.0 Hz, 1H, 5′‐O*H*_adenosine_), 5.01 (dd, *J* = 6.2, 2.9 Hz, 1H, 3′‐C*H*_adenosine_), 4.22–4.16 (m, 3H, 4′‐C*H*_adenosine_ and 5‐C*H*_*2*‐pent‐1‐yn‐yl_), 3.56 (dt, *J* = 11.5, 5.3 Hz, 1H, 5′‐C*H*_adenosine_), 3.47 (ddd, *J* = 11.5, 6.9, 5.5 Hz, 1H, 5′‐C*H*_adenosine_), 2.65 (t, *J* = 7.2 Hz, 2H, 3‐C*H*_*2*‐pent‐1‐yn‐yl_), 2.16 (p, *J* = 7.2 Hz, 2H, 4‐C*H*_*2*‐pent‐1‐yn‐yl_), 1.50 (s, 3H, C(C*H*_3_)_2_), 1.31 (s, 3H, C(C*H*_3_)_2_). ^13^C NMR (151 MHz, DMSO‐*d*_6_): δ (ppm) = 159.7 (*C*−6_purine_), 155.9 (*C*−6_adenosine_), 154.1 (*C*−4_purine_), 153.5 (*C*−2_adenosine_), 149.4 (*C*−2_purine_), 148.1 (*C*−4_adenosine_), 143.1 (*C*−8_purine_), 133.0 (*C*−8_adenosine_), 123.4 (*C*−5_purine_), 118.8 (*C*−5_adenosine_), 113.2 (*C*(CH_3_)_2_), 96.5 (*C*−2_pent‐1‐yn‐1‐yl_), 90.0 (*C*−1′_adenosine_), 86.7 (*C*−4′_adenosine_), 82.2 (*C*−2′_adenosine_), 81.6 (*C*−3′_adenosine_), 70.4 (*C*−1_pent‐1‐yn‐1‐yl_), 61.6 (*C*−5′_adenosine_), 42.3 (*C*−5_pent‐1‐yn‐1‐yl_), 27.3 (*C*−4_pent‐1‐yn‐1‐yl_), 27.1 (C(*C*H_3_)_2_), 25.2 (C(*C*H_3_)_2_), 16.1 (*C*−3_pent‐1‐yn‐1‐yl_). FTIR (neat): $\tilde{v}$ (cm^−1^) = 2978 (C─H), 1604 (C=C/C=N), 1562 (N─H_bend_), 1377 (C─N), 1157, 1080 (C─O).

To the protected nucleoside (51 mg, 0.09 mmol, 1 eq.), 7 N NH_3_ in CH_3_OH (1 mL) was added and the reaction mixture was stirred at 50°C in a sealed tube for 2 h. The solvent was evaporated under reduced pressure and the residue was dissolved in H_2_O/CH_3_OH (1:1, 3 mL) and Dowex resin (H^+^ form, 50 mg) was added. The mixture was stirred at 50°C for 5 h. Next a saturated solution of NaHCO_3_ was added at room temperature, and the mixture was stirred for 30 min. The excess resin was removed by filtration and the filtrate was evaporated under reduced pressure. The residue was purified by flash chromatography (CH_3_OH/CH_2_Cl_2_ = 20:80; Ø = 1 cm, *l* = 10 cm), and product **22** was obtained as an off‐white solid (12 mg, 0.02 mmol, 26%). *R*_*f*_ = 0.46 (10/90 CH_3_OH/CH_2_Cl_2_). mp = 153.9°C. Purity (HPLC *C*): > 99% (*R*_*t*_ = 10.1 min). Exact mass (ESI): m/z = calcd. for C_20_H_22_^35^ClN_10_O_4_ [M + H^+^] 501.1509, found 501.1534. ^1^H NMR (600 MHz, DMSO‐*d*_6_): δ (ppm) = 8.16 (s, 1H, 8‐C*H*_purine_), 8.15 (s, 1H, 2‐C*H*_adenosine_), 7.57 (s, 1H, N*H*_2*‐*adenosine_), 6.90 (s, 1H, N*H*_2‐purine_), 5.97 (d, *J* = 6.8 Hz, 1H, 1′‐C*H*_adenosine_), 5.59 (s, 1H, 5′‐O*H*_adenosine_), 5.46 (s, 1H, 2′‐O*H*_adenosine_), 5.22 (s, 1H, 3′‐O*H*_adenosine_), 4.99 (td, *J* = 5.5, 1.3 Hz, 1H, 2′‐C*H*_adenosine_), 4.22–4.18 (m, 2H, 3′‐C*H*_adenosine_ and 5‐C*H*_*2*‐ pent‐1‐yn‐yl_), 3.99 (td, *J* = 3.9, 2.3 Hz, 1H, 4′‐C*H*_adenosine_), 3.68 (dd, *J* = 12.2, 3.8 Hz, 1H, 5′‐C*H*_adenosine_), 3.53 (dd, *J* = 12.3, 4.1 Hz, 1H, 5′‐C*H*_adenosine_), 2.63 (t, *J* = 7.2 Hz, 1H, 3‐C*H*_*2*‐ pent‐1‐yn‐yl_), 2.15 (p, *J* = 7.2 Hz, 1H, 4‐C*H*_*2*‐ pent‐1‐yn‐yl_). ^13^C NMR (151 MHz, DMSO‐*d*_6_): δ (ppm) = 159.7 (*C*−6_purine_), 156.0 (*C*−6_adenosine_), 154.1 (*C*−4_purine_), 153.1 (*C*−2_adenosine_), 149.4 (*C*−2_purine_), 148.3 (*C*−4_adenosine_), 143.2 (*C*−8_purine_), 133.8 (*C*−8_adenosine_), 123.4 (*C*−5_purine_), 119.1 (*C*−5_adenosine_), 96.1 (*C*−2 _pent‐1‐yn‐1‐yl_), 89.4 (*C*−1′_adenosine_), 86.7 (*C*−4′_adenosine_), 71.6 (*C*−2′_adenosine_), 71.0 (*C*−3′_adenosine_), 70.7 (*C‐*1‐_pent‐1‐yn‐1‐yl_), 62.2 (*C*−5′_adenosine_), 42.3 (*C*−5_pent‐1‐yn‐1‐yl_), 27.2 (*C*−4_pent‐1‐yn‐1‐yl_), 16.1 (*C*−3_pent‐1‐yn‐1‐yl_). FTIR (neat): $\tilde{v}$ (cm^−1^) = 3318 (O─H/N─H), 2928 (C─H), 2241 (C≡C), 1612 (C=C/C=N), 1562 (N─H_bend_), 1331 (C─N), 1026 (C─O).

#### 4.1.21 Synthesis of (2*R*,3*S*,4*R*,5*R*)−2‐({4‐[4‐(2‐Amino‐6‐Chloro‐9*H*‐Purin‐9‐yl)Butyl]−1*H*−1,2,3‐Triazol‐1‐yl}Methyl)−5‐(6‐Amino‐9*H*‐Purin‐9‐yl)Tetrahydrofuran‐3,4‐diol (**23**)

Alkyne derivative **12** (100 mg, 0.40 mmol, 2 eq.) and 5′‐azido‐5′‐deoxyadenosine (**18**, 59 mg, 0.20 mmol, 1eq.) were dissolved in dry EtOH (1.5 mL) under N_2_ atmosphere. Copper(II) acetylacetonate (3 mg, 0.012 mmol, 0.06 eq.) and sodium ascorbate (24 mg, 0.12 mmol, 0.6 eq.) were added to the reaction mixture, and the mixture was stirred at room temperature for 2 days. The mixture was filtered through Celite and the solvent was evaporated. The residue was purified by flash chromatography (CH_3_OH/CH_2_Cl_2_ = 30:70; Ø = 2 cm, *l* = 15 cm) and product **23** was obtained as an off‐white solid (44 mg, 0.08 mmol, 40%). *R*_*f*_ = 0.83 (30/70 CH_3_OH/CH_2_Cl_2_). mp = 149.6°C. Purity (HPLC *C*): 100% (*R*_*t*_ = 10.6 min). Exact mass (ESI): m/z = calcd. for C_21_H_25_^35^ClN_13_O_3_ [M + H^+^] 542.1886, found 542.1913. ^1^H NMR (600 MHz, DMSO‐*d*_6_): δ (ppm) = 8.19 (s, 1H, 8‐C*H*_adenosine_), 8.14 (s, 1H, 2‐C*H*_adenosine_), 8.12 (s, 1H, 8‐C*H*_purine_), 7.67 (s, 1H, 5‐C*H*_triazole_), 7.30 (s, 2H, N*H*_2*‐*adenosine_), 6.88 (s, 2H, N*H*_2*‐*purine_), 5.89 (d, *J* = 5.2 Hz, 1H, 1′‐C*H*_adenosine_), 5.58 (d, *J* = 5.9 Hz, 1H, 2′‐O*H*_adenosine_), 5.43 (d, *J* = 5.0 Hz, 1H, 3′‐O*H*_adenosine_), 4.73–4.59 (m, 3H, 2′‐C*H*_adenosine_ and 5′‐C*H*_*2‐*adenosine_), 4.26–4.20 (m, 2H, 3′‐C*H*_adenosine_ and 4′‐C*H*_adenosine_), 4.04 (t, *J* = 7.1 Hz, 2H, 4‐C*H*_*2‐*butyl_), 2.59–2.55 (m, 2H, 1‐C*H*_*2‐*butyl_), 1.78 (p, *J* = 7.3 Hz, 2H, 3‐C*H*_*2‐*butyl_), 1.49 (p, *J* = 7.6 Hz, 2H, 2‐C*H*_*2‐*butyl_). ^13^C NMR (151 MHz, DMSO‐*d*_6_): δ (ppm) = 159.7 (*C*−6_purine_), 156.1 (*C*−6_adenosine_), 154.0 (*C*−4_purine_), 152.7 (*C*−2_adenosine_), 149.3 (*C*−2_purine_), 149.3 (*C*−4_adenosine_), 146.3 (*C*−4_triazole_), 143.3 (*C*−8_purine_), 139.8 (*C*−8_adenosine_), 123.3 (*C*−5_purine_), 122.6 (*C*−5_triazole_), 119.2 (*C*−5_adenosine_), 87.8 (*C*−1′_adenosine_), 82.3 (*C*−4′_adenosine_), 72.6 (*C*−2′_adenosine_), 70.9 (*C*−3′_adenosine_), 51.1 (*C*−5′_adenosine_), 42.7 (*C*−4_butyl_), 28.5 (*C*−3_butyl_), 25.9 (*C*−2_butyl_), 24.3 (*C*−1_butyl_). FTIR (neat): $\tilde{v}$ (cm^−1^) = 2928 (C─H), 1639, 1609 (C=C/C=N), 1566 (N─H_bend_), 1053 (C─O).

#### 4.1.22 Synthesis of (2*R*,3*S*,4*R*,5*R*)−2‐({4‐[3‐(2‐Amino‐6‐Chloro‐9*H*‐Purin‐9‐yl)Propyl]−1*H*−1,2,3‐Triazol‐1‐yl}Methyl)−5‐(6‐Amino‐9*H*‐Purin‐9‐yl)Tetrahydrofuran‐3,4‐diol (**24**)

Alkyne derivative **13** (100 mg, 0.42 mmol, 2 eq.) and 5′‐azido‐5′‐deoxyadenosine (**18**, 62 mg, 0.21 mmol, 1eq.) were dissolved in dry EtOH (1.5 mL) under N_2_ atmosphere. Copper(II) acetylacetonate (3 mg, 0.012 mmol, 0.06 eq.) and sodium ascorbate (25 mg, 0.13 mmol, 0.6 eq.) were added, and the mixture was stirred at room temperature for 2 days. The mixture was filtered through Celite, and the solvent was evaporated. The residue was purified by flash chromatography (CH_3_OH/CH_2_Cl_2_ = 30:70; Ø = 2 cm, *l* = 14 cm) and product **24** was obtained as an off‐white solid (19 mg, 0.04 mmol, 17%). *R*_*f*_ = 0.74 (30/70 CH_3_OH/CH_2_Cl_2_). mp = 115.3°C. Purity (HPLC *C*): 100% (*R*_*t*_ = 10.1 min). Exact mass (ESI): m/z = calcd. for C_20_H_23_^35^ClN_13_O_3_ [M + H^+^] 528.1730, found 528.1757. ^1^H NMR (600 MHz, DMSO‐*d*_6_): δ (ppm) = 8.21 (s, 1H, 8‐C*H*_adenosine_), 8.14 (s, 1H, 2‐C*H*_adenosine_), 8.12 (s, 1H, 8‐C*H*_purine_), 7.75 (s, 1H, 5‐C*H*_triazole_), 7.29 (s, 2H, N*H*_2*‐*adenosine_), 6.89 (s, 2H, N*H*_2*‐*purine_), 5.90 (d, *J* = 5.2 Hz, 1H, 1′‐C*H*_adenosine_), 5.59 (d, *J* = 5.8 Hz, 1H, 2′‐O*H*_adenosine_), 5.45 (d, *J* = 5.0 Hz, 1H, 3′‐O*H*_adenosine_), 4.75–4.61 (m, 3H, 2′‐C*H*_adenosine_ and 5′‐C*H*_*2‐*adenosine_), 4.27–4.21 (m, 2H, 3′‐C*H*_adenosine_ and 4′‐C*H*_adenosine_), 4.07 (t, *J* = 7.1 Hz, 2H, 3‐C*H*_2*‐*propyl_), 2.56 (t, *J* = 8.0 Hz, 2H, 1‐C*H*_2*‐*propyl_), 2.10–1.99 (m, 2H, 2‐C*H*_2*‐*propyl_). ^13^C NMR (151 MHz, DMSO‐*d*_6_): δ (ppm) = 159.7 (*C*−6_purine_), 156.1 (*C*−6_adenosine_), 154.1 (*C*−4_purine_), 152.7 (*C*−2_adenosine_), 149.3 (*C*−2_purine_), 149.2 (*C*−4_adenosine_), 145.5 (*C*−4_triazole_), 143.3 (*C*−8_purine_), 139.8 (*C*−8_adenosine_), 123.4 (*C*−5_purine_), 122.8 (*C*−5_triazole_), 119.2 (*C*−5_adenosine_), 87.8 (*C*−1′_adenosine_), 82.3 (*C*−4′_adenosine_), 72.6 (*C*−2′_adenosine_), 70.9 (*C*−3′_adenosine_), 51.2 (*C*−5′_adenosine_), 42.6 (*C*−3_propyl_), 28.6 (*C*−2_propyl_), 22.1 (*C*−1_propyl_). FTIR (neat): $\tilde{v}$ (cm^−1^) = 3325 (O─H/N─H), 2978 (C─H), 1609 (C=C/C=N), 1562 (N─H_bend_), 1134, 1049 (C─O).

### 4.2 Pharmacological/Biological Assays

#### 4.2.1 Cloning and Mutagenesis

Genes encoding for SAS1 and SAS2 were prepared according to the previously described method [9].

#### 4.2.2 Protein Production and Purification

SAS1 and SAS2 were purified according to the previously described method [9].

#### 4.2.3 Assaying the Inhibitory Effect of Compounds **8a–8c**, **9a–9q** on SAS1 and SAS2

A total of 0.5 µM of enzyme (SAS1 or SAS2) was incubated in a total volume of 10 µL in a reaction buffer containing 100 mM Hepes⋅Na (pH 7.5), 200 mM NaCl, 20 mM MgCl_2_, and 20 mM KCl in the presence of 5 mM ATP, 1 mM GDP, and 2 mM of the respective inhibitor in DMSO for 5 min at 30°C. The reactions were stopped by addition of 300 µL chloroform and brief (15 s) heating to 95°C. (Phases were separated by centrifugation (10 min, 4°C, 17,300*g*). The aquaeous phase was subjected to HPLC measurements performed with an Agilent 1100 Series system (Agilent Technologies) and an anion exchange column (Metrosep A Supp 5–150/4.0). After running for 25 min with a buffer containing 100 mM (NH_4_)_2_CO_3_ (pH 9.25), the reaction product ppGpp was detected at a wavelength of 260 nm. The conversion of GDP and ATP to ppGpp and AMP was plotted against a reaction without inhibitor utilizing Microsoft Excel and GraphPad Prism 8.

#### 4.2.4 Assaying the Inhibitory Effect of Compounds **19–24** on SAS1 and SAS2

To a mixture of 1 mM each GDP, ATP, and respective inhibitors in double‐distilled H_2_O, 0.2 µM enzyme was added and incubated for 60 min at 30°C. 40 µL double‐distilled H_2_O was added to 10 µL of this reaction, immediately followed by 150 µL of chloroform. After brief shaking (5–10 s), heating to 95°C for 15 s, and flash‐freezing in liquid nitrogen, the tubes were thawed again and centrifuged (17,300*g*, 10 min, 4°C). The aqueous phase was analyzed by HPLC on an Agilent 1260 Infinity system (software: ChemStation Rev. B04.03‐SP1) equipped with a Metrosep A Supp 5–150/4.0 column (Metrohm). Nucleotides were separated with an isocratic flow (0.6 mL min^−1^) of 100 mM (NH_4_)_2_CO_3_ (pH 9.25) and detected at 260 nm wavelength. The conversion of GDP and ATP to ppGpp and AMP was plotted against a reaction without inhibitor utilizing Microsoft Excel and GraphPad Prism 8.

#### 4.2.5 Concentration‐Response Curve of Compound **21** on SaSAS1

Different concentrations of compound **21** (0, 0.1, 1.0, 5.0, and 16 µM) were prepared in double‐distilled H_2_O, and their inhibitory effects were measured as described previously. The conversion of GDP and ATP to ppGpp and AMP was plotted against a reaction without inhibitor utilizing Microsoft Excel and GraphPad Prism 8.

### 4.3 Computational Modeling

First, receptor and ligand atoms were processed. Therefore, the experimental structure with the PDB ID 6EWZ [7] was loaded into the Molecular Operating Environment (MOE) v2024.0601 [12]. Chain B of the homodimer was kept. The ligand structures were adjusted by deleting the phosphate groups of AMPCPP and GTP and the ribose of GTP. This way, only the guanine and adenosine moieties were retained. Water molecules > 4.5 Å away from the ligands were also deleted.

Then, the linker between the guanine and adenosine was constructed using the “Builder” tool and the guanine was modified to match the structure of **21**. First, the ligand atoms were selected and followed by an energy minimization of the selected atoms. Beforehand the partial charges were adjusted to the AMBER:EHT forcefield as implemented in MOE. Next, the selected ligand atoms were again energy minimized, but this time these atoms and the receptor atoms within an 8 Å radius were set to “Tether,” allowing a deviation of 0.5 Å. All other atoms in the system were kept inert. The steric plausibility of the resulting binding mode was assessed using the “Mogul Geometry Check” [13] as implemented in the Hermes software suite. First, the ligand was adjusted to match the atom naming and bond conventions used in the Cambridge Structural Database (CSD) [14]. Then, the check was conducted using standard settings. No major problems with bonds, angles, and torsions were detected.

For the binding site comparison between SaSAS1/2 and BsSAS1/2, the following data were used:

**Table:** 

Name	Uniprot ID	Structure ID	Synonyms	Reference
SaSAS1	W8TMV6	AlphafoldDB ID AF‐W8TMV6‐F1‐v6	YjbM, relP	Jumper et al. 2021 [15]
SaSAS2	W8U368	PDB ID 6EWZ	ywaC	Manav et al. 2018 [7]
BsSAS1	O31611	PDB ID 5F2V	RelQ, YjbM	Steinchen et al. 2015 [8]
BsSAS2	P39583	PDB ID 6FGK	RelP, YwaC	Steinchen et al. 2018 [9]

## Conflicts of Interest

Oliver Koch is a scientific advisor at Nuvisan ICB GmbH and Prosion GmbH.

## Data Availability

The data that support the findings of this study are available from the corresponding author upon reasonable request.
